# Mechanical Model of Geometric Cell and Topological Algorithm for Cell Dynamics from Single-Cell to Formation of Monolayered Tissues with Pattern

**DOI:** 10.1371/journal.pone.0126484

**Published:** 2015-05-14

**Authors:** Sëma Kachalo, Hammad Naveed, Youfang Cao, Jieling Zhao, Jie Liang

**Affiliations:** 1 Department of Bioengineering, The University of Illinois at Chicago, Chicago, IL, 60607; 2 Computer, Electrical and Mathematical Sciences and Engineering Division, King Abdullah University of Science and Technology, Thuwal, Saudi Arabia; Centrum Wiskunde & Informatica (CWI) & Netherlands Institute for Systems Biology, NETHERLANDS

## Abstract

Geometric and mechanical properties of individual cells and interactions among neighboring cells are the basis of formation of tissue patterns. Understanding the complex interplay of cells is essential for gaining insight into embryogenesis, tissue development, and other emerging behavior. Here we describe a cell model and an efficient geometric algorithm for studying the dynamic process of tissue formation in 2D (e.g. epithelial tissues). Our approach improves upon previous methods by incorporating properties of individual cells as well as detailed description of the dynamic growth process, with all topological changes accounted for. Cell size, shape, and division plane orientation are modeled realistically. In addition, cell birth, cell growth, cell shrinkage, cell death, cell division, cell collision, and cell rearrangements are now fully accounted for. Different models of cell-cell interactions, such as lateral inhibition during the process of growth, can be studied in detail. Cellular pattern formation for monolayered tissues from arbitrary initial conditions, including that of a single cell, can also be studied in detail. Computational efficiency is achieved through the employment of a special data structure that ensures access to neighboring cells in constant time, without additional space requirement. We have successfully generated tissues consisting of more than 20,000 cells starting from 2 cells within 1 hour. We show that our model can be used to study embryogenesis, tissue fusion, and cell apoptosis. We give detailed study of the classical developmental process of bristle formation on the epidermis of *D. melanogaster* and the fundamental problem of homeostatic size control in epithelial tissues. Simulation results reveal significant roles of solubility of secreted factors in both the bristle formation and the homeostatic control of tissue size. Our method can be used to study broad problems in monolayered tissue formation. Our software is publicly available.

## Introduction

*Cell theory* postulates that cell is the building block of an organism. It also assumes that the behavior of an organism is the sum of the actions of individual cells that constitute the organism (see [1] for detailed review of this once widely accepted theory). In contrast, the *organismal theory* treats the organism as a whole, rather than looking at its individual parts, *i.e.* cells. Several studies have shown that mutations that affect the size or shape of individual cells can change the size and shape of the organ, as seen in plant leaf [2, 3]. However, it was also shown that there exists cooperation between leaf cells at some level, suggesting the existence of an organismic response [1, 3, 4].

How different tissue patterns arise mechanistically is an important question. Experimentally, it is challenging to design and conduct studies to identify specific effects of different attributes of individual cells and cell-cell interactions on cellular pattern formation. Computational studies can complement experimental studies in providing important insight. A number of computational methods have already been developed [5–12].

Among these, the cellular Potts model is a widely used method for studying cell behavior, where a lattice site can be a square, a triangle, or a hexagon. Each cell is modeled as a collection of about 25–50 lattice sites [13]. Cells have a predefined size, and neighboring cells interact with specific binding energy, which mimics effects of the underlying biology, *e.g.*, cadherin interactions [14]. Cellular Potts model can be used to study pattern formation involving multiple cells. For example, Käfer *et al* studied cell packing using a Potts model on a set of 4 cells [15]. They concluded that both cell adhesion and cortex contractility determines cell patterning in the *Drosophila* retina. Merkes *et al* further carried out a detailed study of contact inhibited chemotaxis in controlling *de novo* and sprouting blood vessel growth [14].

However, cell shape and topology are not modeled directly in the cellular Potts model. Extensive post-processing is often required for more realistic cell shapes. In addition, the underlying forces for cell movement are not explicitly accounted for. Changes such as growth and division of cells are not modeled directly, as they are based on Metropolis moves of flips of the identities of boundary lattice sites bordering two cells. Cell motions are achieved through energy minimization after stochastic fluctuations of flips of lattice sites introduced by Metropolis moves. Due to these requirements, it is difficult to use Potts model to study details of cell proliferation and cell migration, as such details are not adequately captured by collection of lattice sites and by flipping these lattice sites. Another obstacle towards more realistic cell shape is the computational cost. As more lattice sites are required for detailed geometry of a cell, the computational cost grows rapidly if a tissue of many cells is to be modeled realistically. To study such problems, parallel computing is often necessary [16].

A different class of cell models based on the finite element method have also been developed [17–21]. While they provide very realistic descriptions of cell shapes, they have inflexible boundary conditions and cannot model dynamic changes in cell shape. For example, it is difficult to study cell growth, cell migration, cell birth, and cell apoptosis using finite element based models [17, 18].

The center-based model (*e.g.*, as in the implementation of the CellSys system) approximates each cell by an isotropic, elastic, and adhesive sphere [22]. Cells can interact with each other and can respond to environmental stimuli. Growth, division, and cell migration can all be modeled. It can be used to model large tissues containing many cells. This model is specifically designed to study details of pair-wise cell forces based on an idealized model, *i.e.*, cellular interactions can be treated as interactions between homogeneous elastic sticky spheres, as in the JKR model [22]. No detailed descriptions of cell shapes are included, and any shape deviation from sphere (*e.g.*, polygon) is ignored [22]. Furthermore, cells after division are assumed to take the form of spherical shape immediately. As a result, center based models are not well-suited to study details of the dynamic changes in cell shape and in cell topology during the growth process. They also cannot be used to study biological problems that deviate from the idealized model, such as increased tension on the interface of two cell populations (*e.g.* tumor cells *vs* normal cells), as the shape of the cell-cell interaction interface is not taken into account.

Vertex models are another class of very successful models. They are based on the postulation that cell shape is determined by minimizing the energy under forces acting on cell junctions, which are represented as vertices. Designed to study packing and remodeling of epithelial cells [23, 24], in which the apical areas of a layer of epithelial cells are modeled, vertex models incorporate changes due to cell division and cell extrusion. They can incorporate properties of cell size, shape, and elasticity, and can be used to study cell birth, growth, migration, and apoptosis at varying degrees. They have been used widely to study tissue morphology [25], tissue dynamics [26, 27], wound closure [28], cell sorting [29], regulation of cell division and growth [30], and the genesis of cell polarity [31].

However, cell shapes in existing vertex models are not modeled with sufficient detail. For examples, cells are always polygonal and do not have curved boundaries. Cell growth is also not modeled in detail. In addition, initial conditions require a plural number of cells (*e.g.*, 16 cells), often with periodic boundary conditions. Cell death can only be modeled for the special case associated with a specific type of topological change [24, 32]. Furthermore, the apical areas of cells in the epithelial tissue cannot be simply interpreted as cell sizes, since it is assumed that changes in the apical area is accompanied by concomitant flow of cytosol in the apical region towards the basal region of the cell. Cell height may therefore increase, with possibly overall little change in cell volume [24, 30, 32]. Because of such model choices, the apical area of a cell can shrink and even disappear without significantly changing the cell volume [24, 30, 32]. Tissue growth is realized primarily through cell divisions [23]. The growth of individual cell that do not conform to the average apical area is also not modeled explicitly.

Chimeric methods such as the viscoelastic cell model by Jamali et al. [33] and the immersed boundary framework by Rajniak [34] can model realistic cell shape, cell growth, cell division, cell motion, and cell-cell interactions. However, these methods are unsuitable for simulating large tissues due to the model choice of representing the shape of a cell by a network of linear Voigt elements or by a collection of boundary points connected through linear springs, which leads to substantial computational overhead.

Here we describe a new dynamic cellular model that accounts for cell size, shape, and interactions between cells in 2D. Individual cells can be added when born, or removed when dead. Cells can also grow or shrink in size. They can divide and interact with each other, with specifics dictated by cell types and cellular micro-environments [35, 36].

We aim to develop a general modeling framework for simulating cell growth and tissue pattern in epithelial tissue development. We use three simplified examples of biological studies to highlight the novel aspects of our model: (1) tissue development from a single or a very small number of cells, without the requirement of the initial condition of a population of cells and without periodic boundary conditions; (2) geometric and topological changes when two population of cells fuse together by closing spatial gaps; and (3) more realistic model of cell deaths. These are discussed in the context of studying embryogenesis, tissue fusion, and cell apoptosis.

While existing methods can address partially the three issues that motivate our studies, there is not a single model, except the one presented in this study, which can simultaneously address fully all these issues. Specifically, (1) most of existing models rely on strict initial conditions and/or boundary conditions. For example, vertex models usually require an initial tissue of at least 16 cells, and cannot simulate tissue development starting from a single cell. This prevents their applications in studying the initial stages of tissue development that involves only a handful of cells. Furthermore, many vertex models require periodic boundary conditions, which make it difficult to study realistically tissue development with heterogeneous patterns. The subcellular finite element models also have the same problems. (2) Most of existing models cannot be used to model realistically important events in tissue patterning such as tissue fusion, which involves significant geometric and topological changes of cell shapes and connectedness among cell populations. This is conceptually an important event and technically a challenging problem that is often underappreciated. To our knowledge, no existing vertex models and subcellular finite element models can model the important fusion process of cells and tissues. (3) Upon cell apoptosis, extensive geometric changes occur in the apoptotic cell and its neighbors. None of the vertex and finite element models can model this process realistically. For example, the important process of cell apoptosis is modeled as simple extrusion process in vertex models. In contrast, our model gives full account of cell size reduction and ultimately its elimination due to DNA fragmentation and cytoplasm shrinkage, which are modeled through decreased surface pressure or cell removal from the tissue [37]. (4) Most of the existing methods are computationally inefficient and cannot simulate the biological relevant number of cells in a tissue. Further summary of the strengths of the present model can be found in Table 1.

**Table 1:** Comparisons to existing methods.

Methods	Our Model	Cellular Potts Model	Subcellular Finite Element Methods	Center-based Models	Current Vertex Models	Immersed Boundary Framework	Viscoelastic cell Model
Flexible cell size	**Yes.**	Pre-defined	**Yes.**	**Yes.**	**Yes.**	**Yes.**	**Yes.**
Realistic cell shape	**Yes.** Cell interior is represented as polygon and the exposed boundary as curved edge	**Indirectly.** Extensive post-processing is often required for more realistic cell shapes	**No.** Rigid and inflexible boundary conditions; Cannot model dynamic changes in cell shapes	**No.** All cells are represented as **pre-defined sphere**. Not suited for studying dynamic changes in cell shape.	**No.** All cells are represented as **polygon**.	**Yes.** Cell is represented by a collection of boundary points connected through linear springs.	**Yes.** Cell is represented by a network of linear Voigt elements.
Cell growth and division	**Yes.** By adding partition wall (division plane) in the dividing cell.	**Indirectly.** Based on Metropolis moves of flips of the identities of boundary lattice sites bordering two cells.	**Yes.** By dividing the sub elements into halves.	**Yes.** By adding one more sphere as a daughter cell.	**Limited.** Tissue Growth is realized primarily through cell divisions. The growth of individual cell that do not conform to the average apical area is not modeled explicitly.	**Yes.** By introducing a point source inside the cell that creates a fluid flow causing the cell to grow by pushing its boundaries and increasing its area/volume.	**Yes.** By adding partition wall (division plane) in the dividing cell.
Cell death	**Yes.**	**Yes.**	**Yes.**	**Yes.**	Only in a special case associated with a topological change.	**Yes.**	**Yes.**
Cell motions	**Yes.** Achieved through surface energy minimization after forces are added on vertices.	**Indirectly** achieved through energy minimization after stochastic fluctuations in flips of lattice sites introduced by Metropolis moves.	**Yes.** Achieved through intra-cellular elements velocity potential minimization after the cell velocity is changed.	**Yes.** Achieved through pre-defined energy setup, and then re-building of the Voronoi diagram.	**Yes.** Achieved through energy minimization after forces are added on vertices.	**Yes.** Achieved through energy minimization after the forces are added on vertices.	**Yes.** Achieved through protrusion, adhesion, and contraction.
Independence of initial conditions	**Yes.**	**Yes.**	**Yes.**	**Yes.**	**No.** Plural number of cells are required (*e.g.* 16)	**Yes.**	**Yes.**
Independence of boundary conditions	**Yes.**	**Yes.**	**No.** hard-wall boundary conditions.	**Yes.**	**No.** Periodic boundary conditions.	**Yes.**	**Yes.**
Interactions between cells	**Yes.** Through the mutual vertex and edge between cells.	**Limited.** Shapes and maximum neighbors are pre-defined.	**Yes.** Intra-cellular edges between sub elements are added between cells as interactions.	**Limited.** Shape of interaction interface is not taken into account.	**Yes.** Through the mutual vertex and edge between cells.	**Yes.** Through the mutual vertices between cells.	**Yes.** Through the mutual vertices between cells.
Tissue fusion	**Yes.**	**Yes.**	**Yes.**	**Yes.**	**No.**	Not described but possible in principle.	Not described but possible in principle.
Computational Efficiency	**High**	**High**	**Low**	**High**	**Medium**	**Medium**	**Medium**

We also give two more detailed examples of how this computational cell model can be used: 1) to verify or refute mechanism proposed to explain the formation of various tissue patterns, and 2) to investigate effects of inhibition range of secreted factors and cell division type on the homeostatic size control of the olfactory epithelium tissue. In 1), we study the long standing problem of bristle formation on the epidermis of fruit fly *D. melanogaster*. We discuss simulation results of bristle formation in *D. melanogaster* following several existing models for expression of bristle formation related genes *achaete (ac)*, *Delta (Dl)*, and *Notch (N)* from literature. We further explore the relationship between the inhibition field radius (due to the solubility of Delta-like protein) and the width of the stripe (where the genes *ac* and *Dl* are highly expressed) through simulations based on our model. In 2), we study the fundamental problem of the homeostatic size control of the olfactory epithelium tissue based on a model of stem cells, progenitor cells, and differentiated cells. We discuss the effects of the inhibition range of secreted factors from differentiated cells in controlling tissue size. In addition, we assess the importance of symmetric and asymmetric division of stem cells in tissue size control.

This paper is organized as follows. We first describe details of the cell model, along with the data structure for its implementation. We then describe the mechanical forces used in our model. This is followed by simulation studies of changes in cell geometry and cell rearrangement due to these forces. For biological applications, we discuss the examples of embryogenesis, tissue fusion, and apoptosis, followed by more detailed simulation studies on the bristle formation phenomenon on the epidermis of fruit fly *D. melanogaster*. We then discuss the effects of diffusion radius of Delta-like protein and the width of the stripes of highly expressed *ac*, *Dl*, and *N* genes, as well as studies on the homeostatic size control of epithelial tissues.

## Materials and Methods

### Geometric Model of Cell

Similar to existing vertex models, the underlying physics of our two-dimensional cellular model is that of a well-studied topic of surface energy minimization, also called *bubble formation*[38–40]. In this model, each cell minimizes its surface energy, as prominently observed in the developing retina of *Drosophila*, where differential expression of N-cadherin leads to the formation of an overall shape that minimizes their surface contact with surrounding cells [41]. There are known exceptions to the assumption of minimal contact energy. For example, during the dorsal closure, the two dimensional epithelium has cell boundaries with non-constant curvatures [42]. Therefore, our method cannot be used to study detailed cell boundary changes when the assumption of minimal contact energy is not valid.

In our two-dimensional model, an isolated cell is idealized and takes up the shape of a disk. When two cells make contact, their common boundary is represented as a line segment. A cell can make contacts with multiple neighboring cells. If a cell still has one or more free boundaries remaining, these boundaries take the shape of arcs. A cell takes the shape of a polygon when it is fully surrounded by other cells. This is similar to previous studies [17, 18, 20, 21].

**Fig 1:**
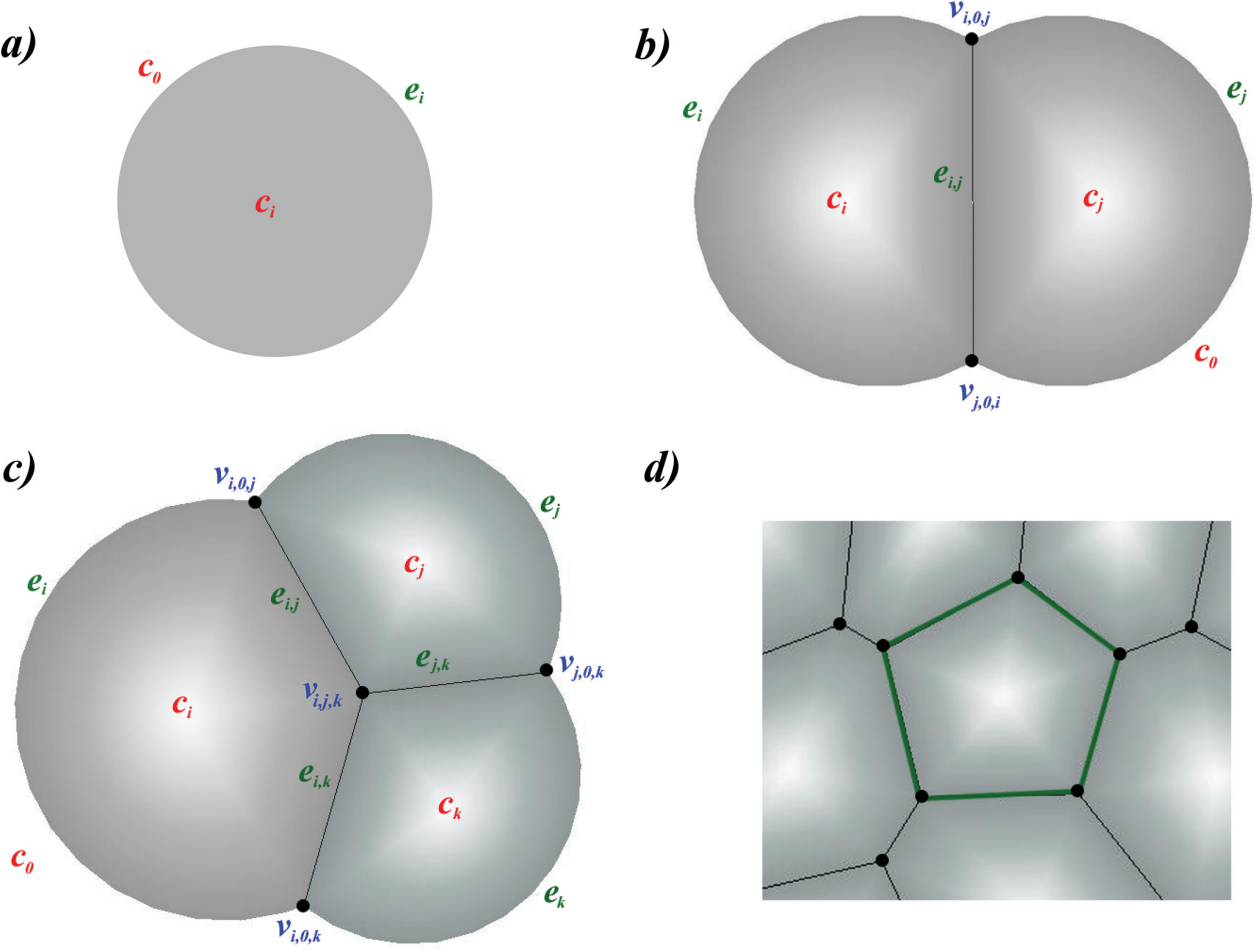
Two-dimensional cell model. a) An isolated cell is modeled as a disk. b) A cell is modeled as a disk segment when contacting other cell(s). An *outer edge* ***e***_*i*_ is an arc or a circle, representing the boundary between cell ***c***_*i*_ and the outside medium (denoted as ***c***_0_). An *inner edge* ***e***_*i*,*j*_ occurs when a cell ***c***_*i*_ is in contact with another cell ***c***_*j*_. Their shared boundary is modeled as a straight line segment. When two cells ***c***_*i*_ and ***c***_*j*_ make contact, their outer edges (arcs) ***e***_*i*_ and ***e***_*j*_ intersect at two vertices ***v***_*i*,0,*j*_ and ***v***_*j*,0,*i*_, which are also the two end-points of the inner edge ***e***_*i*,*j*_. c) When three cells ***c***_*i*_,***c***_*j*_ and ***c***_*k*_ intersect, they form a vertex ***v***_*i*,*j*,*k*_. d) A cell completely surrounded by other cells is represented as a polygon.

Formally, a biological cell is represented by the combination of three types of geometric elements (Fig 1). First, a *geometric cell ***c***_*i*_* is a spatial region representing the volume of cell *i* (Fig 1a). Cells can have different sizes. A cell is a disk when in isolation, but can be a disk segment when the cell is contacting other cell(s). It can take the shape of the union of a polygon and one or more disk segment(s), when there are multiple contacting cells in the surroundings, and at the same time there is one or more free boundaries (Fig 1b and 1c). When completely surrounded by other cells, it takes the form of a polygon (Fig 1d).

Second, *edges* represent the boundaries of a cell. There are two types of edges: *outer edges* ***e***_*i*_ for cell *i* and *inner edges* ***e***_*i*,*j*_ between cell *i* and cell *j* (Fig 1b). An *outer edge* ***e***_*i*_ is an arc or a circle, and represents the boundary between cell ***c***_*i*_ and the outside medium (denoted as ***c***_0_). That is, ***e***_*i*_ = ***c***_*i*_∩***c***_0_ ≠ *ϕ*. ***e***_*i*_ is a full circle when the cell exists in isolation, but becomes one or more arcs if the medium does not fully surround the cell. An *inner edge* ***e***_*i*,*j*_ occurs when a cell ***c***_*i*_ is in contact with another cell ***c***_*j*_, namely, when ***e***_*i*,*j*_ = ***c***_*i*_∩***c***_*j*_ ≠ *ϕ*. Their shared boundary is a face with constant surface curvature [38], but is modeled as a straight line segment here, as the curvature is usually small. An inner edge appears twice, once for each of the neighboring cell, with the order of the two indices reversed. This reflects the fact that each cell has a separate wall.

When a cell interacts with multiple cells, its boundary may contain one or more (possibly disconnected) outer edges, along with one or more (possibly disconnected) inner edges. Overall, the cell boundary forms a closed curve, with straight line segments (inner edges) and/or arcs (outer edges) as component pieces.

Third, *vertex* is the junction point of three edges. When two cells ***c***_*i*_ and ***c***_*j*_ make contact (Fig 1b), their outer edges (arcs) ***e***_*i*_ and ***e***_*j*_ intersect at two vertices, ***v***_*i*,0,*j*_ (indices in clockwise direction) and ***v***_*j*,0,*i*_, which are also the two end-points of the inner edge ***e***_*i*,*j*_ (Fig 1b). When three cells ***c***_*i*_, ***c***_*j*_ and ***c***_*k*_ intersect (Fig 1c), they form a single vertex ***v***_*i*,*j*,*k*_. In our two-dimensional model, we assume no more than three cells can intersect as seen in soap bubbles [38–40]. That is, no more than three edges can meet at a vertex.

For a tissue consisting of *n* cells, we denote the set of cell centers as ***Z*** = {*z*_1_,⋯,*z*_*n*_}, where *z*_*i*_ ∈ ℝ^2^ is the coordinates of the center of cell *i*. The set of edges is denoted as ***E*** ≡ {***e***_*i*_}∪{***e***_*i*,*j*_}, and the set of vertices is denoted as ***V*** = {***v***_*i*,*j*,*k*_}. The overall state ***S*** of a tissue with *n* cells is defined as: ***S*** = (***Z***,***E***,***V***). It fully determines the geometric pattern of the tissue formed by these *n* cells.

### Data Structure

We have used the *HalfEdge* data structure [43] for algorithm implementation.

#### Cell

An object is used to represent a cell, which contains attributes for the biology of the cell, such as cell type, and attributes for visualization, such as color. In addition, a cell object contains a pointer to a boundary edge if the cell is not an isolated cell. Otherwise, the edge-pointer is set to null for a cell in isolation. In the latter case, the radius of the cell is stored.

#### Half-Edge

**Fig 2:**
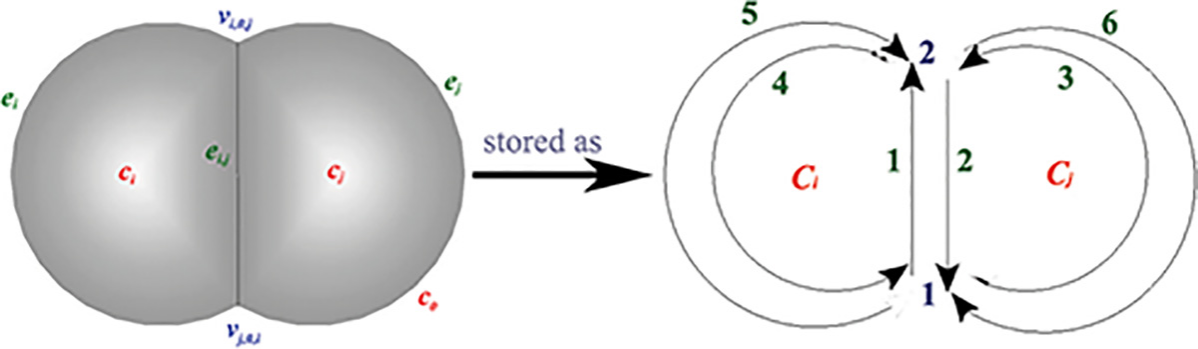
Data Structure. The boundary of a cell is formed by connected edges. It is modeled as an oriented closed curve in the counterclockwise direction. Each physical inner edge is represented twice using two half-edges, once each in opposite directions for each of the two contacting cells. Each physical outer edge is also represented twice with two half-edges, once for the cell, and once for the outside space.

The boundary of a cell is formed by connected edges. We model this boundary as an oriented closed curve in counterclockwise direction. Each physical inner edge is represented twice using two half-edges, once each in opposite directions for each of the two contacting cells. Each physical outer edge is also represented twice with two half-edges, once for the cell, and once for the outside space (Fig 2).

The data structure of a half-edge is an object that contains a pointer to the cell. For an outer edge, this pointer is set to null. It also contains pointers to the two end-vertices. In addition, a pointer to the reverse half-edge is also provided. A next pointer leads to the next half-edge along the counter-clockwise direction of the closed curve. The half-edge data structure also stores the angle of the arc for the outer edges.

#### Vertex

The vertex object simply stores the (*x*,*y*) coordinates and contains a pointer to one of the half edges that starts from this vertex.

#### List of Neighboring Cells

One of the most important advantages of the *Half-Edge* data structure is the access to the neighbors of a cell in *O*(MAX_NEIGHBORS) and maintenance of the list of neighboring cells in *O*(1) time. The neighboring cells can be traversed with the help of reverse edges. A summary of our data structure and the pseudo-code for traversing neighboring cells can be found in S1 Appendix.

Our data structure offers an important advantage in maintaining the list of neighboring cells. As the edges/reverse edges are updated constantly by the topological changes during the growth process, there is no additional cost in terms of time or memory in maintaining the list of neighboring cells. Such easy access and maintenance is only possible in grid-based models with the compromise of lack of details of cell shape and size. In other models, a list of neighboring cells needs to be maintained and updated, for each cell. When the tissue size is large, this maintenance is both time and storage consuming.

### Physical Model of Cell and Cell Growth

#### Stationary Model

We model cells with the assumption that they are in a stationary state, in which changes are slow, and all forces in the system at every moment are balanced out by each other.

We use discrete time steps to model incremental changes of cell volumes, which are dictated by the underlying biology, *e.g.* cell birth, cell death, cell growth and shrinkage, and changes of cell wall properties. In our model, we assume the energy of the cells reaches a minimum at the state ***S***:

E(S)=E(X,E,V)=min.

Forces exerting on the system of cells at state ***S*** have a zero net sum. We model the mechanical forces using only the vertices. The geometry of the whole system, including the edges and cells, and the forces in each cell, all will follow once the vertex set ***V*** is specified. We have:

$$F\left(S\right)\equiv F\left(V\right)=\frac{dE(V)}{dV}=0.$$

#### Mechanical Forces

There are many physical forces that exist in a cell. Cytoskeletal microfilament [44–46], intermediate filaments [47], and cell membrane all exert *compression forces* on a cell. In addition, there exists adhesion or alternatively repulsion force between cells. These forces can be summed up and modeled as a *tension force* that exerts along the direction ***e***_*i*,*j*_ of inner edge (interior cell boundary), or along the tangent direction of outer edge ***e***_*i*_ (free cell boundary).

There are also *expansion forces* in a cell. These include those from microtubules [45, 46, 48, 49] and the extracellular matrix (ECM) [50]. We model these forces as a *pressure force* that acts along the direction normal to the edge. Pressure force only exists for inner edge, not for arc/outer edge, as the internal pressure is compensated by pressure due to the curvature of the arc of the cell boundary. Tension force exists at vertices due to both inner and outer edges.

In our model, cells minimize their energy [41]. In this sense, cells take upon the appearance and behavior of soap bubbles. According to the soap bubble model, cell walls take the shape of constant mean curvature (CMC) surfaces under fixed volume and pressure conditions [38]. The most notable examples are spherical shape and biconcave erythrocyte shape [51]. We therefore model cells as intersecting circular disks.

**Fig 3:**
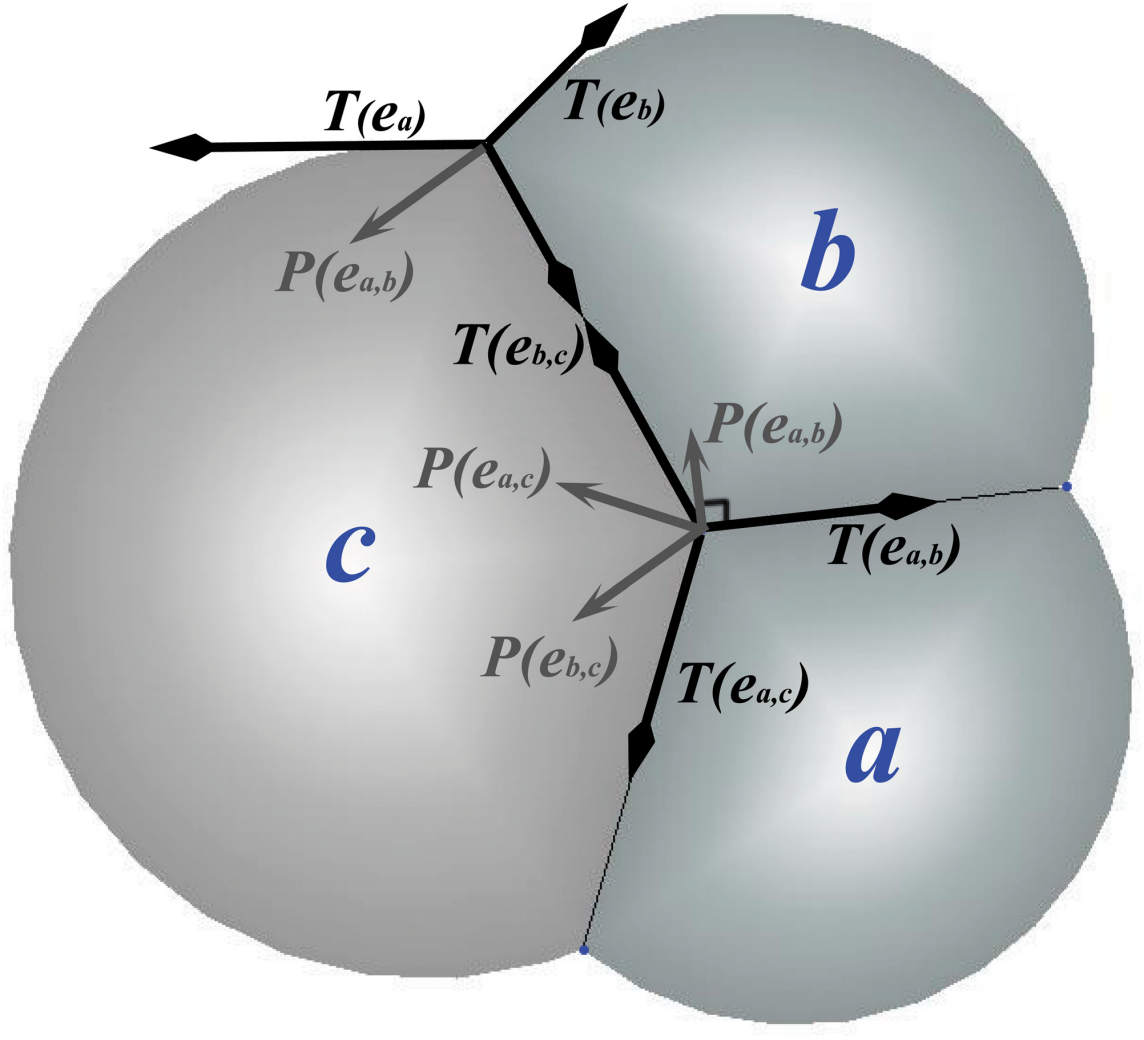
Tension force and pressure. Forces applied to the junction vertex of three cells *a*, *b*, and *c*. The *tension force* ***T***(***e***_*i*,*j*_) exerts along the direction ***e***_*i*,*j*_ of an inner edge (interior cell boundary), or along the tangent direction of outer edge ***e***_*i*_ (free cell boundary), where (*i*,*j*) are the two indices of cells *a*,*b*, or *c*. The *pressure force* ***P***(***e***_*i*,*j*_) acts along the direction normal to the cell boundary, in the direction from the cell with higher pressure to that with lower pressure.

Physical forces are modeled to act at vertices (Fig 3). For inner edges, although the physical cell wall will adopt a curved surface under the bubble model, the curvature is small and we simplify it as a straight line segment. The physical forces originally tangent to the curved surface are now decomposed into two components for the straight line segment. They are the tension force ***T***(***e***_*i*,*j*_) and the pressure force ***P***(***e***_*i*,*j*_). Tension force acts in the direction of shortening the edge, and pressure force follows the direction of the difference of pressure in two cells sharing the edge.

For outer edges, we take the curved surface into account and model it as an arc. In this case, the physical force tangent to the curved surface is modeled directly as the tension force ***T***(***e***_*i*_) for this cell, in the direction of shortening the arc. The pressure inside the boundary cell containing this outer edge determines the curvature of the outer edge, and therefore determines the tangential direction of the tension force.

Formally, the forces applied to a vertex *v* in ***V*** can be decomposed as

$$\begin{array}{c} F_{v}=\sum_{e,\;\text{s.t.}v\in e}\left[T\left(e\right)+P\left(e\right)\right], \end{array}$$ (1)

which sums over all edges ***e***’s with the vertex *v* as an end point. Here **T**(***e***) and **P**(***e***) are the forces acting on edge ***e*** through cell wall tension and intracellular pressure, respectively.

For the edge ***e***_*i*,*j*_ between cells *i* and *j*, the tension force is always tangential to the edge ***e***_*i*,*j*_:

$$T\left(e_{i,j}\right)=\eta \left(i,j\right)e_{i,j},$$

where *η* is the tension coefficient, which may depend on the cell types of both cells, and ***e***_*i*,*j*_ is the edge vector. We assume ***e***_*i*,*j*_ is in the direction of shortening ***e***_*i*,*j*_, otherwise, we add a coefficient “−1” in front of this formula.

Pressure induced force is in the direction normal to the inner edge:

$$P\left(e_{i,j}\right)=\left(P_{i}-P_{j}\right)\left|e_{i,j}\right|n_{\left(i,j\right)},$$

where *P*_*i*_ and *P*_*j*_ are the pressures in two cells, ∣***e***_*i*,*j*_∣ is the length of the inner edge, and ***n***_(*i*,*j*)_ is the unit vector normal to the edge in the direction from the cell with higher pressure to the cell with lower pressure. Although pressure force exerts on the whole inner edge, we decompose it equivalently to the two end vertices, each distributed with $\frac{1}{2}$ of the total pressure force ***P***(***e***_*i*,*j*_).

For an outer edge ***e***_*i*_ of cell *i*, the tension force acting on a vertex ***v*** is always tangential to the arc ***e***_*i*_, in the direction **t**_*v*_ of shortening ***e***_*i*_:

$$T\left(e_{i}\right)=\eta \left(i,0\right)\left|e_{i}\right|t_{v}.$$

Here 0 denotes the outside medium, *η*(*i*,0) is the tension coefficient of the outer edge of cell *i*. The internal pressure is compensated by pressure due to the curvature of the arc of the cell boundary. Therefore, pressure induced force is zero. The value of the pressure inside an outer cell *i* itself is determined by the curvature of the edge:

$$P_{i}=\eta \left(i,0\right)/r_{i}.$$

Here *r*_*i*_ is the radius of the arc.

**Fig 4:**
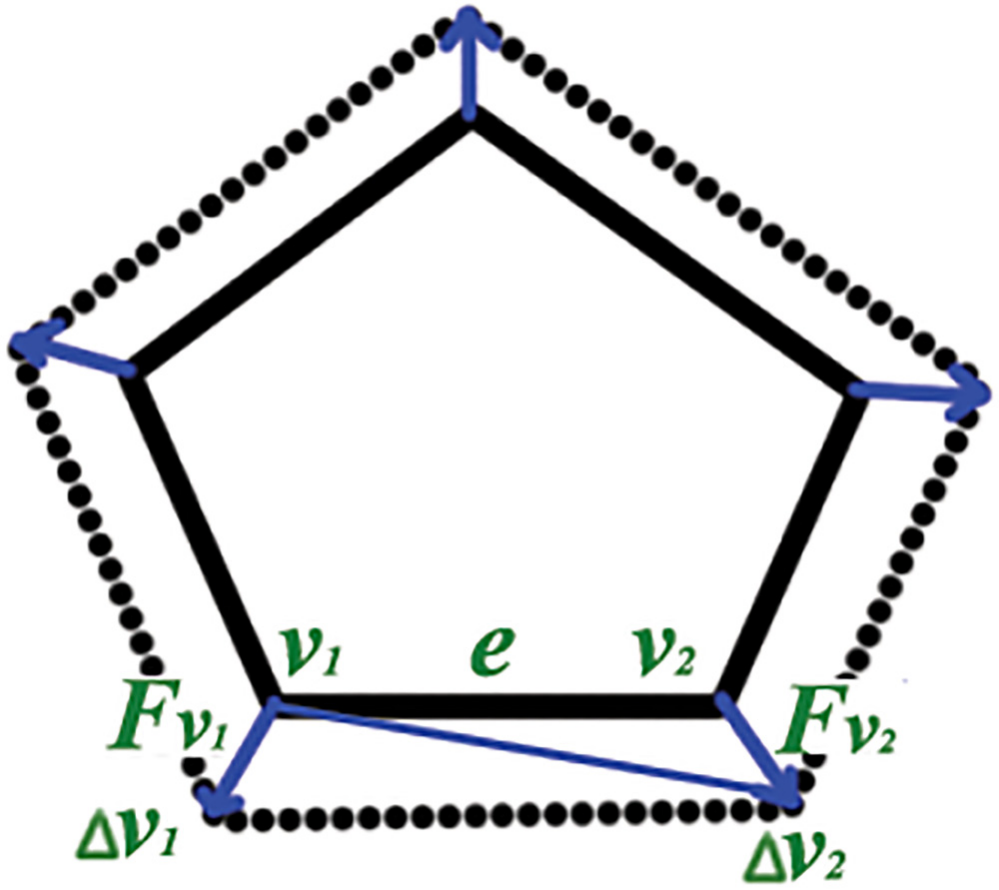
Change in Volume. Diagram representing the forces involved during cell growth. Vertices *v*_1_ and *v*_2_ are moved by Δ*v*_1_ and Δ*v*_2_ due to the forces **F**_*v*_1__ and **F**_*v*_2__, during the growth process respectively. ***e*** is the edge connecting the two vertices. Its length is ∣***e***∣.

In general, forces at the two vertices of an edge can be in different direction, which can result in displacement of the edge. The movement of an edge is the result of the volume change of the cell. The new position of the edge forms an irregular quadrilateral with the edge before the movement. We distribute this volume change to the two vertices, each with a triangle. This volume change happens in each time step (Fig 4).

#### Growth, Shrinkage and Cell Movement

We assign different volume changes to individual cells to reflect different stage of cell cycle. For cell *i*, when Δ*V*_*i*_ > 0, cell *i* grows. When Δ*V*_*i*_ < 0, cell *i* shrinks. When Δ*V*_*i*_ = 0, cell *i* stays in a steady state.

#### Cell Shape

**Fig 5:**
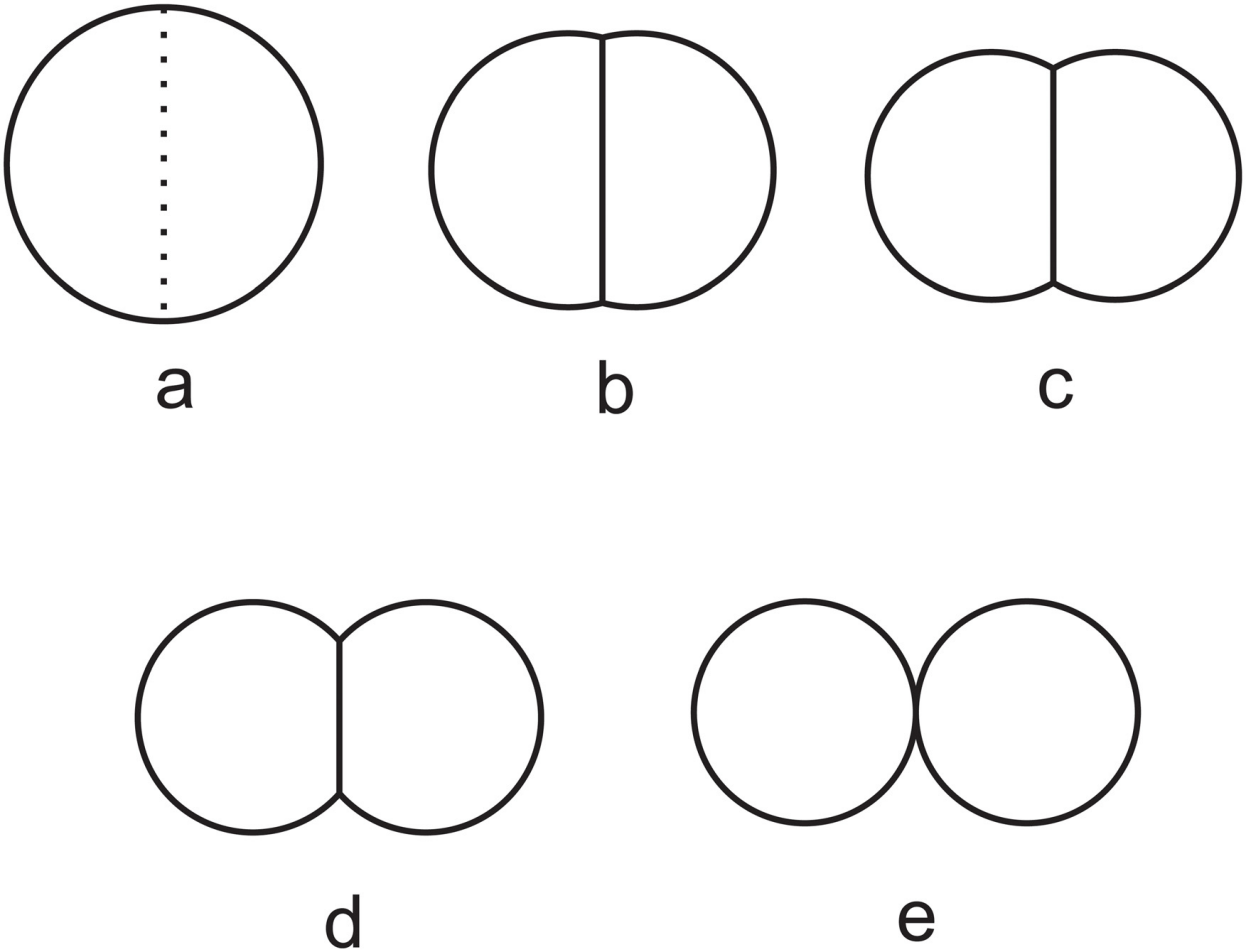
Cell geometry. Cell geometry is determined by the ratio of the tension coefficients. a) when *η*(*i*,*j*) = 0, there is no tension on an inner edge, and it can be regarded as an imaginary cell wall; b) when *η*(*i*,*j*) = 0.5*η*(*i*,0), there is a strong adhesion force between the two cells; c) when *η*(*i*,*j*) = *η*(*i*,0) = *η*(*j*,0), the two cells behave as if physically they have the same wall; d) when *η*(*i*,*j*) = 1.5*η*(*i*,0), there is a weak adhesion force between the two cells; e) when *η*(*i*,*j*) ≥ 2*η*(*i*,0), The two cells have no adhesion and behave like soccer balls. Adding an inner wall would be more costly, as it is equivalent to adding two outer walls. In this case, the overall energy of the two cells is not reduced.

In our model, the cell walls take the shape of constant mean curvature surfaces under fixed volume and pressure conditions. Physically, each cell has its own wall, and the surface tension *η*(*i*,*j*) at an inner edge depends on the properties of both cell walls. The final shape of a cell depends on the ratio of tension coefficient *η*(*i*,*j*) for inner edge and *η*(*i*,0) for the outer edge (Fig 5). When *η*(*i*,*j*) = 0, there is no tension on an inner edge, and it can be regarded as an imaginary cell wall. When *η*(*i*,*j*) = 0.5*η*(*i*,0), there is a strong adhesion force between the two cells. When *η*(*i*,*j*) = *η*(*i*,0) = *η*(*j*,0), the two cells behave as if physically they have the same wall. When *η*(*i*,*j*) ≥ 2*η*(*i*,0), adding an inner wall would be more costly, as it is equivalent to adding two outer walls. In this case, the overall energy of the two cells is not reduced. The two cells therefore have no adhesion and behave like soccer balls (Fig 5).

#### Calculating Forces

For cell *i* that experiences cell volume change Δ*V*_*i*_ at time step *t*, the net force at each of its vertices can be calculated based on the assumption of stationary state:

$$\begin{array}{c} \Delta V_{i}=\frac{1}{2}\sigma \sum_{e}\sum_{v(e)}\left|F_{v(e)}\times e\right|. \end{array}$$ (2)

Here the coefficient of 1/2 represents the change in volume of one of the two triangles formed by the division of the irregular quadrilateral (Fig 4). The summation is over all edges ***e*** of cell *i* and over both end vertices {*v*(***e***)} of each edge, “×” represents vector cross product, and *σ* is the constant for the integration step. The derivation of Eq (2) can be found in S1 Appendix. Eq (2) is solved to obtain the forces at each vertex.

#### Updating vertex position

At time *t*+1, the location of a vertex ***v***_*i*_ after the volume change is updated to:

$$\begin{array}{c} v_{i}\left(t+1\right)=v_{i}\left(t\right)+\sigma F_{v_{i}}\left(t\right), \end{array}$$ (3)

where ***v***_*i*_(*t*) and ***v***_*i*_(*t*+1) are locations of vertex *i* before and after the time step, respectively. Time *t* is an integer representing the number of time steps since the initial time, *σ* is a constant that controls the convergence rate towards stationary state, and ***F***_v_*i*__ is the net force exerting at vertex location ***v***_*i*_ (see S1 Appendix for derivation of Eq (3)).

#### Algorithm for Calculating Stationary State

We assume all cells exist in stationary state at the end of each time step of simulation. Cells can grow or shrink during a time step. During each time step, some cells may be in a growth phase, and their volumes increase. Other cells may shrink in size. There may also be cells that maintain constant volumes. The amount of volume changes are assigned from models of underlying biological process. The altered cell volume leads to movement of cell boundaries. In addition, cell wall properties such as the surface tension coefficients may also be different at different time steps.

All these changes are introduced in increments of small fractions. For each increment, we solve Eq (2) to obtain the updated forces. We then move the vertices using Eq (3) to their new locations. After the final increment of volume or cell property change is introduced, we continue iterations with constant volume and cell properties. Vertices are further moved until the system relaxes and reaches stationary state, and a balance of the forces is established (Eq 2). This is the same as applying a gradient search method to find a local minimum of system energy of the cells [52]. We then take the geometric patterns of the cells at this state as that of time step *t* + 1.

The procedure for computing the stationary state of the cell pattern after one time step is illustrated in Table 2. Here **F**_*v*_*i*__ are the forces acting on vertex *i*; **V**(*t*) = (***v***_1_(*t*),⋯,***v***_*m*_(*t*)) is the vector of coordinates of all of the *m* vertices at time *t*; Δ 𝓥(*t*) = (Δ*V*_1_,⋯,Δ*V*_*m*_) is the vector of desired volume changes associated with the vertices for all cells at time *t*, Δ*η*(*t*) is the vector representing desired changes in the cell properties (*e.g.*, cell tension coefficients, cell color) for all cells at time step *t*. The output is the new coordinates of the vertices **V**(*t* + 1) at time step *t* + 1.

**Table 2:** Algorithm 1. UpdateCellPattern (***V***(*t*), Δ 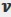
(*t*), Δ*η*(*t*), *σ*, *k*).

//*ε*: Threshold of forces
//*k*: Parameter for step size in incremental volume change.
**while** ***F***_*v*_ > *ε* for any vertices or Δ 𝓥(*t*) not reached yet **do**
Solve Eq 2 to obtain updated forces ***F***_*v*_ for all vertices after updating *η* with Δ*η*(*t*).
**if** desired amount of changes in Δ 𝓥(*t*) not reached yet **then**
Introduce incremental changes Δ 𝓥(*t*)/*k*,
**end if**
Obtain new positions for all vertices using $v_{i}^{\prime }=v_{i}+\sigma F_{v_{i}}$
Update topological changes if required
**end while**
Assign $v_{i}(t+1)=v_{i}^{\prime }$ for all vertices
**return** ***V***(*t* + 1) = (***v***_1_ (*t* + 1), ⋯, ***v***_*m*_ (*t* + 1))

The overall simulation of cellular pattern formation is carried out by repeatedly applying this algorithm to model different biological phenomena, with pre-defined time-dependent volume changes and cell properties changes assigned as input. Stochasticity and other physical factors can be incorporated in schemes that assign these changes.

### Topological Changes of Cellular Pattern

An important ingredient in modeling dynamic changes of cells is an accurate account of all topological changes. We discuss these changes below.

#### Cell Birth, Cell Division, and Cell Death

Topological changes occur during cell birth, cell division, and cell death. In our model, a new cell is generated at cell birth. We model this by inserting a new disk. A new cell is also formed if an existing cell divides. For cell division, we add an edge inside the existing dividing cell and update the cell walls.

We model the process of cell death by gradually decreasing the cell size and eventually removing it completely. This mimics the real process of cell apoptosis, in which the suicide program of apoptosis of a cell leads to fragmentation of the DNA, shrinkage of the cytoplasm, membrane changes and eventual cell death without lysis or damage to neighboring cells [37]. We carry out two primitive operations. First, all outer edges of the dying cell (if they exist) are removed at the moment when cell dies; Second, the inner edges of all the cells contacting the dying cell are replaced with outer edges.

#### Cell Contact Changes

**Fig 6:**
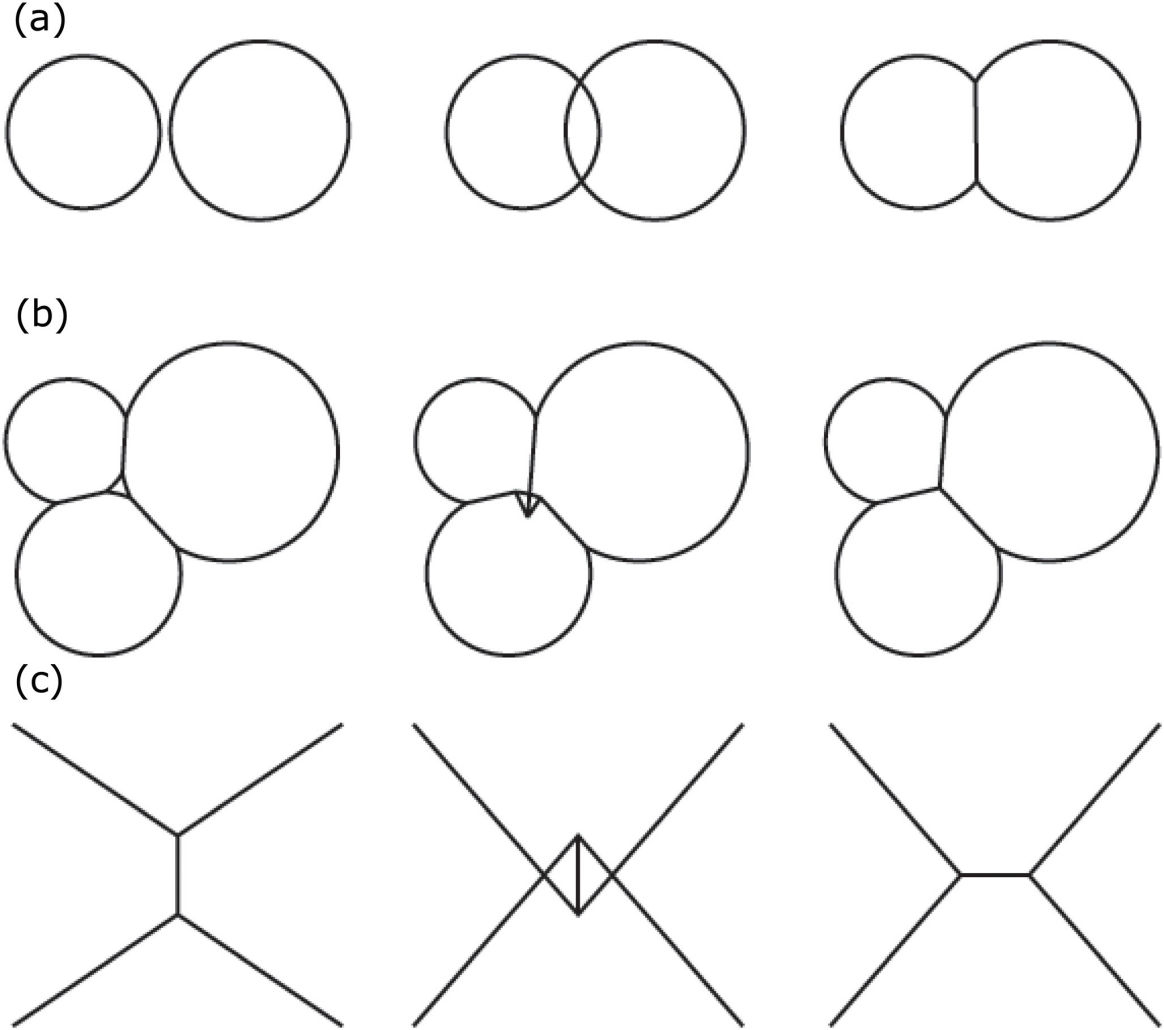
Topological primitives. Possible topological changes when cells grow or when their boundaries move. (a) Edge insertion. When two isolated cells come into contact, we add an edge to represent the intersection plane of the boundary between these two cells. (b) Void removal. When three cells come into contact, the curve triangular empty space is replaced by a vertex where the inner edges meet. (c) Edge flip. When two previously disconnected cells expand to meet while pushing away two previously connected cells, we replace one inner edge with a new inner edge.

In addition to cell birth and cell death, there are three additional types of topological changes when cells grow or shrink and their boundaries move, resulting in cell rearrangement. We use three primitives to model these topological changes, which occur when the same space would be occupied by more than one cell (Fig 6):

#### Edge Insertion

When two cells grow, they may come in contact with each other. When this happens, we add an inner edge to represent the newly formed intersection plane.

#### Void Removal

When three cells are grown together, new inner edges are introduced between two contacting cells. At the moment when three cells meet at a common vertex, we need to replace the curved triangular empty space (void) with a new vertex where the three inner edges meet.

#### Edge Flip

When two originally disconnected cells expand and come into contact, they may squeeze away two previously contacting cells. In this case, we remove the inner edge between two cells originally in contact, and add a new inner edge between the two cells that now come into contact.

Together with the three topological changes of inserting a cell due to cell birth, inserting an inner edge due to cell division, and deleting a cell due to cell death, we exhaust all possible topological changes of cellular patterns modeled in two dimensional space. In our model, these topological changes can occur at any discrete time step during the simulation.

The full account of all possible topological changes allow us to model details of: (1) Cell growth and cell shrinkage, (2) cell division, (3) cell death/apoptosis, (4) fusion events of two-cell, three-cell, and many-cells, (5) cell neighbor swaps associated with edge flips, and (6) void removal or hole filling (Fig 6). Among these, details of (1), (3), and (4) cannot be modeled in current existing vertex models. These new technical developments are essential to study important biological problems that are not easily amenable to other methods, such as embryogenesis, tissue fusion, and cell apoptosis, as discussed in later sections.

### Visualization and Movie Generation Tool

**Fig 7:**
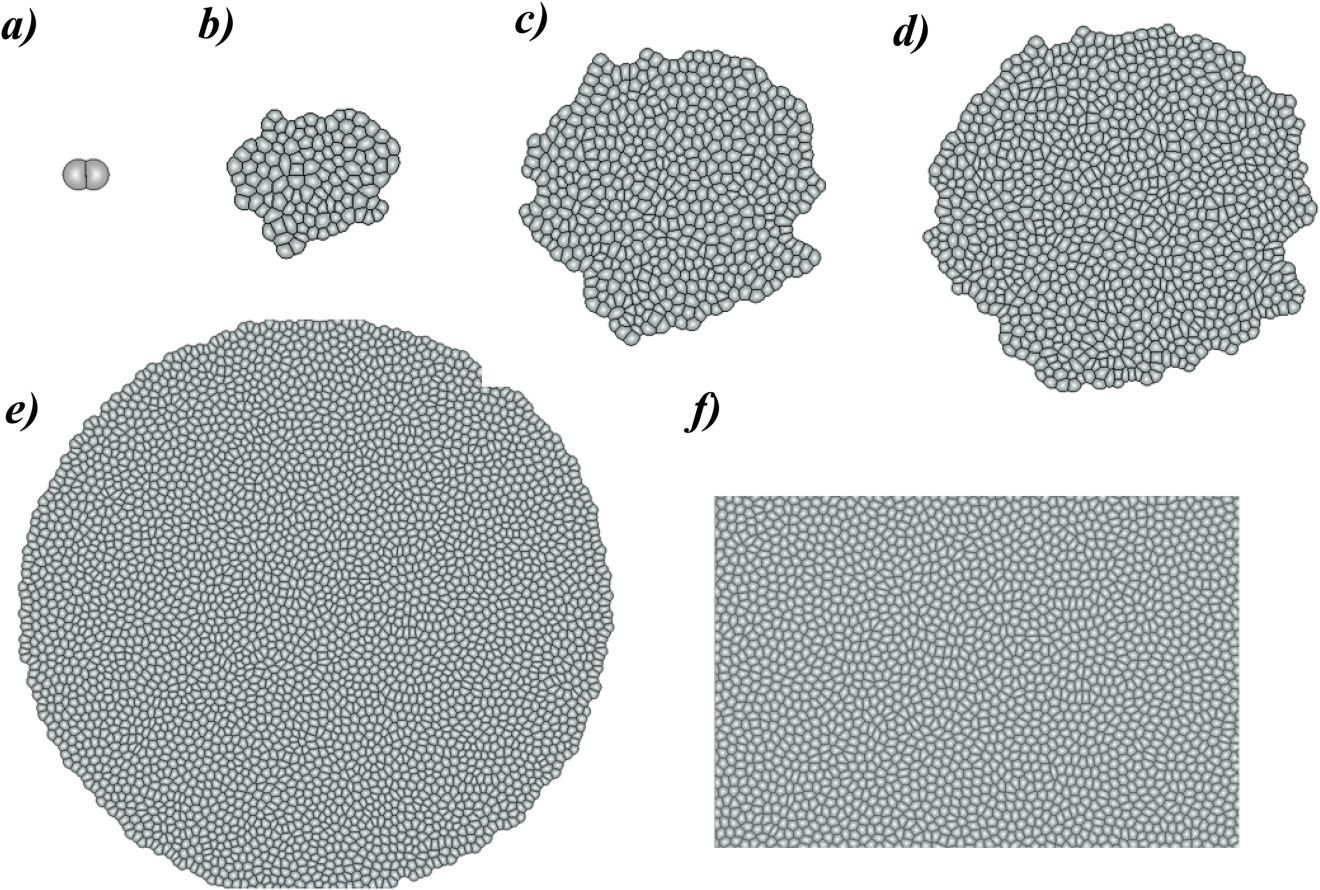
Visualization of the growth of tissue from 2 to 4,000 cells. a) 2 cells; b) 100 cells; c) 500 cells; d) 1,000 cells; e) 4,000 cells, and f) zoomed in view of 4,000 cells.

The overall state **S** of a tissue can be saved to a file at each time step. This can be visually inspected by a visualization and movie generation tool. Application of such a tool allows the user to follow the development of the tissue at every time step. Fig 7 shows the development of a tissue from 2 cells to approximately 4,000 cells. The visualization tool provides options of zooming, altering the background color and saving a particular frame. It also allows the user to generate a movie of the growth process.

### Running Time

Our implementation of the model is efficient and robust. We can grow a tissue from 2 to 1,000, 5,000, or 10,000 cells in approximately 104, 562, or 1,380 seconds, respectively on a Pentium 1.6 GHz processor. This time includes the time to write the state of the tissue at every time step for visual illustration. On average, a tissue of size 12,500 cells can be generated in 30 minutes. We have successfully generated tissues of size up to 20,000 cells, this compares favorably to that of 10,000 cells reported in a previous study [24]. In this previous study, Matlab was used for implementation, which is expected to be considerably slower than our implementation in C++.

## Results

We now describe applications of our cell model and simulation method to illustrate how they can be used to explore mechanisms of cellular pattern formation in nature. We first give three simplified examples.

### Simplified Examples: Embryogenesis, Tissue Fusion, and Apoptosis

#### Embryogenesis and early stage of tissue formation

**Fig 8:**
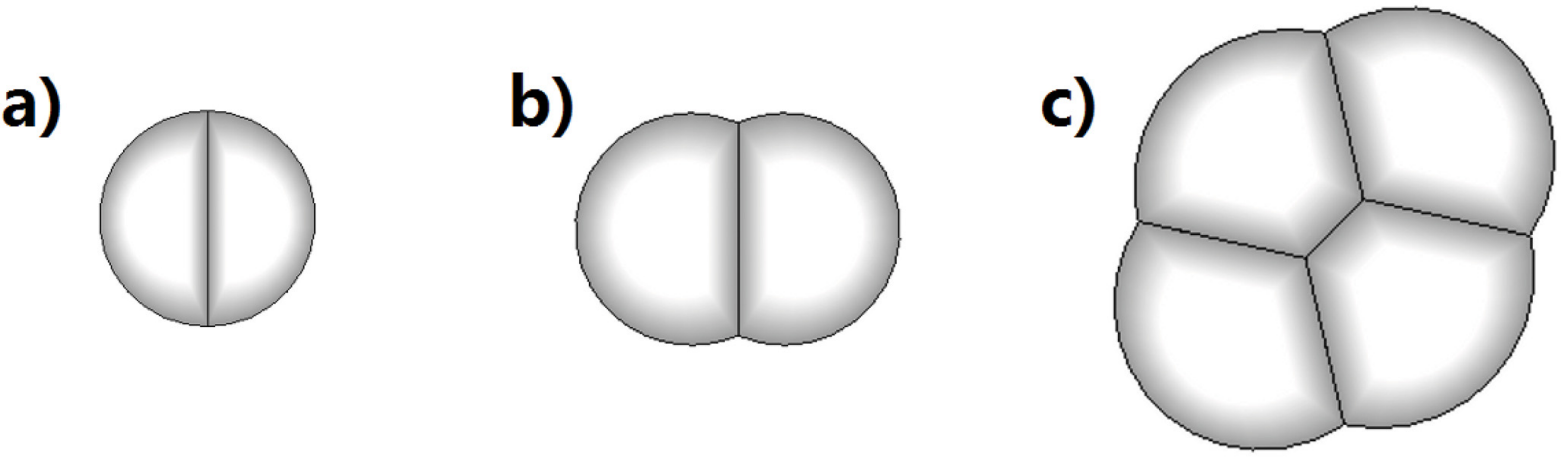
Model of tissue development starting from a single cell. (a) A single cell and its plane of first division; (b) Two daughter cells after the first division, each is slight deformed from its shape in (a); (c) The formation of four cells after two cell divisions.

The early stages of embryogenesis are critically important, as the embryonic stem cell lines are derived during this time. The fertilized egg (day 1) divides to form a 2 cell embryo, followed by subsequent divisions to form 4 cell, 8 cell and so on until a colony of cells is formed by the fourth day. The colony becomes hollow from the middle, forming the blastocyst. Pluripotent embryonic stem cell lines are developed at this stage. The tissues of the embryo start to emerge and the cells become multipotent subsequently. Early embryogenesis is largely monolayered and cell-cell interactions are an important component of this process [53, 54]. Therefore our model is well suited to study this developmental stage. An example of tissue development starting from a single cell is shown in Fig 8 (S1 Movie), which can serve as a toy model for studying the early stage of embryogenesis. Fig 8 shows the process of the first two rounds of division during the growth of a single cell. Fig 8a shows a single cell and the plane of its first division. Fig 8b shows the two daughter cells after a short period of post-division growth. Fig 8c shows the formation of four-cells after the second round of cell division. Note that cell shapes are modeled more realistically and take the form enclosed by spherical arcs and polygonal line segments.

#### Tissue fusion: gap closing

Two or more groups of cells spatially separated may come into contact and become fused together after growth and migration to form a single continuous tissue. In embryonic development, the formation of many organs and tissues, including palate, heart, neural tubes, and eyes, depend on correctly controlled tissue fusion. Malfunction of the fusion events can lead to serious diseases such as cleft palate, spina bifida, and head defects [55]. Tissue fusion is also important for cancer metastasis where cells migrate and invade [56], and for wound healing where tissue remodels [57]. The etiology of these defects are complex and a number of genetic studies have implicated adhesion molecules [58], apoptosis, epithelial-to-mesenchymal transition and cell migration [55]. However, the mechanical basis for the fusion events have not been studied yet. We illustrate with a simple example to demonstrate that some geometric and topological details of the fusion event can be studied using our model.

**Fig 9:**
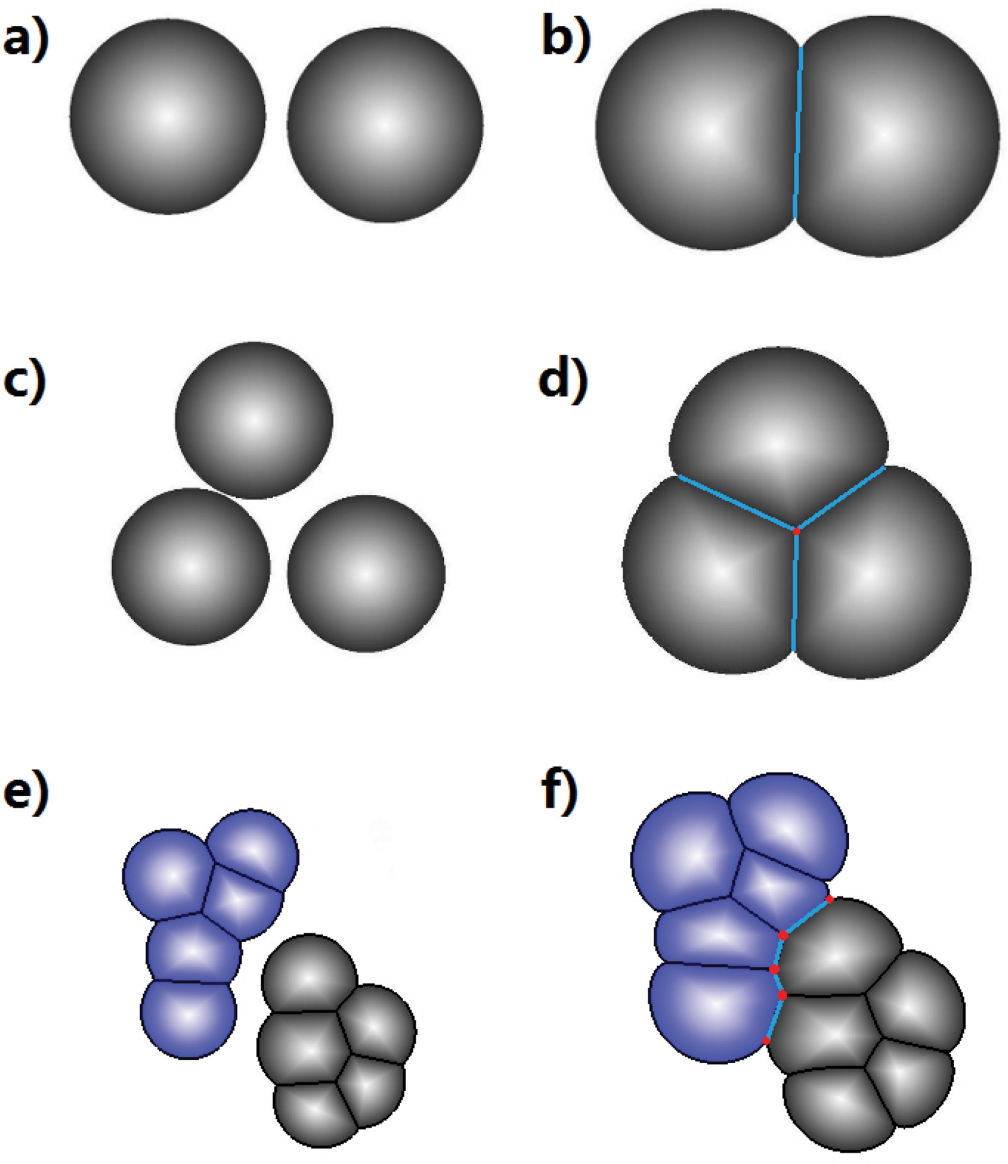
Fusion of cells and tissues. (a) and (b) shows two separate growing cells come into contact and become fused together. A new edge is formed between the two cells. (c) and (d) shows the case of three growing cells fusion. Three new edges and a new vertex are formed after fusion. (e) and (f) shows the fusion of two growing tissues. Two separate tissues contact with each other and become fused together to form a continuous tissue. The new edges and vertices formed are highlighted.

The basic events of tissue fusion are topological contacts. Our model can be used to study details of such fusion events. When two cells grow to form a contact (Fig 9a), a new edge is formed between these two cells (Fig 9b). Upon this contact, both cells become deformed from the idealized shape of sphere, as a result of balancing the pressure forces from the opposing cells (Fig 9b). In our model, three cells may also simultaneously come into contact (Fig 9c). Here new edges are formed between each of the three pairs of neighboring cells, with the additional introduction of a new vertex at the point where the three new edges meet (Fig 9d).

Topological changes in tissue fusion involving many cells are more complex. An example of two growing tissues, each consisting of 5 cells (blue and grey), fusing to form one single tissue of 10 cells is shown in Fig 9e (before) and Fig 9f (after) (S2 Movie). During this fusion process, 4 new edges and three new vertices (highlighted) are formed. These fairly complex sequential events of topological changes can be captured, tracked, and studied in detail using our cell growth model.

#### Apoptosis of peripheral cells

Cell apoptosis [59], or programmed cell death, is essential for many important biological processes, including embryonic development [60] and homeostatic tissue size control [61]. Disrupted apoptosis may lead to diseases, such as neurodegenerative diseases [62] and various types of cancer [62, 63]. Dislodged cells devoid of cellular matrix contact often experience anoikis, a special form of apoptosis [64, 65], and the ability to evade anoikis is an important attribute of metastatic cancer cells [66].

Our model can be used to study apoptosis. Cell death can be explicitly modeled during cell growth and tissue development. The geometric changes in surrounding cells that accompany cell apoptosis can also be modeled realistically. Unlike existing vertex models, programmed cell death does not need to coincide with the topological changes of void removal (also called T2 transition [23]), and can occur anywhere dictated by the underlying model of biology. In contrast, cell death occurs in the vertex model only during T2 transitions [23]), which is only possible for cells in the interior of a tissue fully surrounded by other cells.

**Fig 10:**
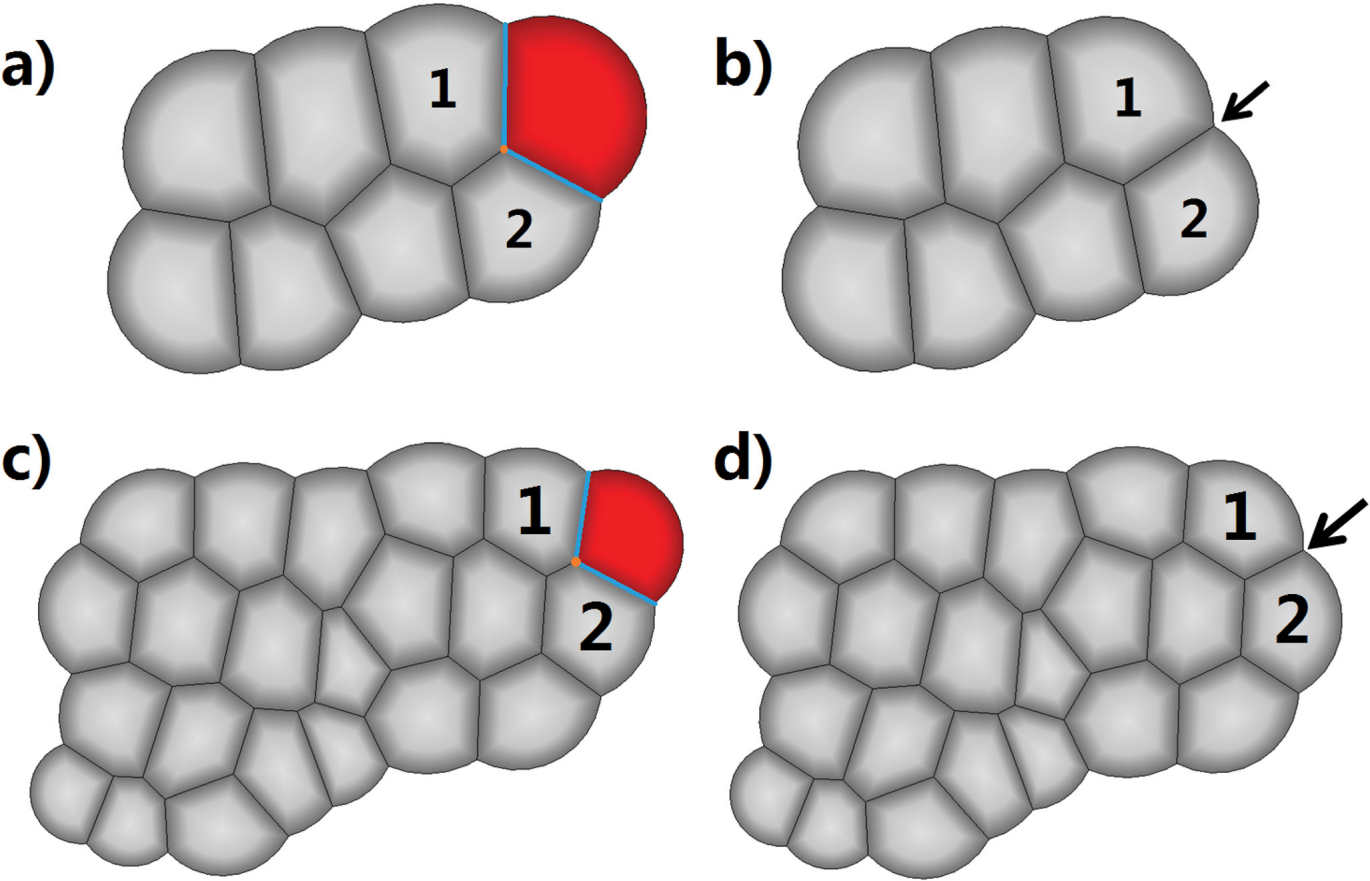
Apoptosis of a peripheral cell. (a) A small tissue before the labeled red cell proceeds to apoptosis. (b) The tissue after the demise of the red cell. The black arrow points to the location where the red cell was. The apoptosis of the red cell caused significant changes on its neighboring cells (labeled 1 and 2). A larger tissue before (c) and after (d) the labeled red cell proceeds to apoptosis. The black arrow in (d) points to the location where the red cell was apoptosized.

Fig 10 shows two examples of cell apoptosis in peripheral tissue (S1 Movie). Fig 10a and 10c each shows a small tissue before a cell (colored in red) becomes apoptosized. Fig 10b and 10d each shows the corresponding tissue after the apoptosis. Here arrows point to the locations where the apoptosis cells were located. Upon cell apoptosis, there are significant changes in cell tension and pressure forces in surrounding cells (labeled with number 1 and 2), resulting in significant changes in cell geometry. Forces on edges of each of the surrounding cells also experience rebalancing. All such changes, including the intermediate states (not shown) and the sequence of events, are explicitly accounted for in our model.

### Bristle Formation

We now give two examples of more detailed studies. We first study a long standing problem, namely, the bristle plotting puzzle. Bristle cells are widely found in different species, including insect epidermis and legs [67]. Bristles are sensor cells, and are important for detecting external stimuli through the rigid exoskeleton [68, 69]. When a bristle is deflected, the pivoting of the shaft in its socket sends a signal to the central nervous system [70, 71]. There is molecular evidence that scales on the butterfly and moth wings are evolutionarily derived from bristles, and their development is governed by similar underlying mechanism as bristle formation in insects [72–74]. Bristle patterning therefore has become an excellent model system in the study of cellular pattern formation.

Here we demonstrate how simulations using our cell model can help explain bristle formation on the epidermal region of *D. melanogaster*. We also discuss our simulation results for the formation of scales on butterfly and moth wings.

#### Models proposed to explain bristle formation

**Fig 11:**
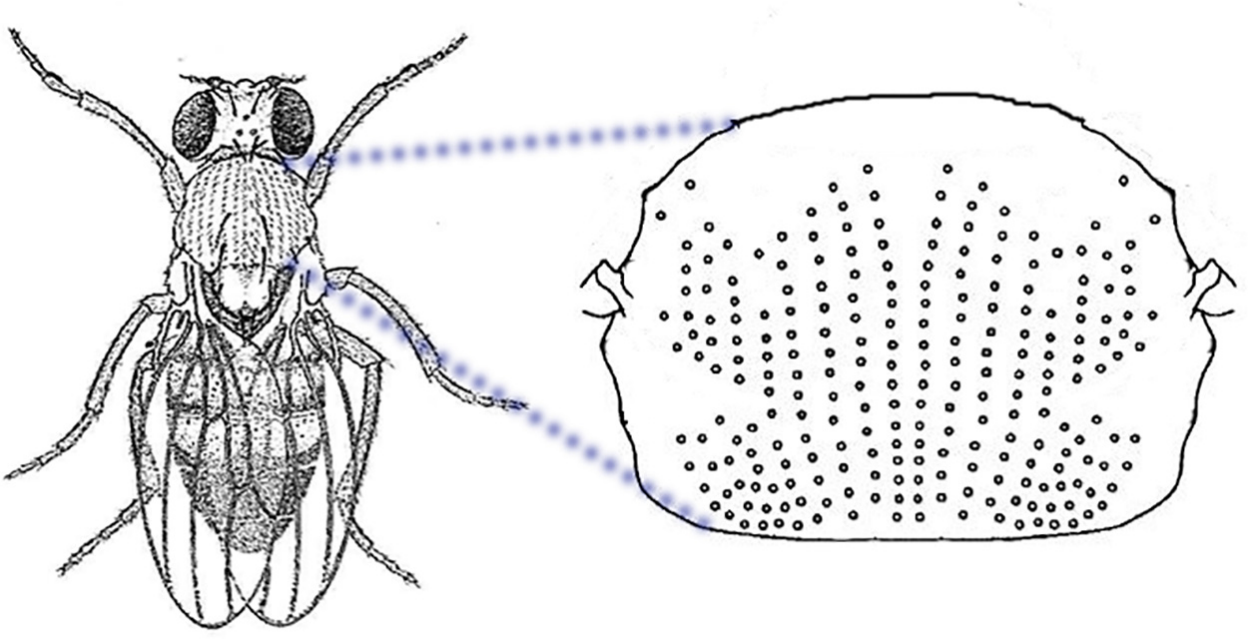
Bristles. Cartoon representation of the bristles on the epidermis of fruit fly. Most bristles are organized in regular rows that are parallel or perpendicular to the body axis or limbs. The bristles are evenly spaced and aligned within each of the rows. Modified from http://www.biology-resources.com/drawing-fruit-fly.html

An adult fruit fly (*D. melanogaster*) has 500,000 cells on its epidermis, but only 1% of them are bristles [75]. The position and number of bristles vary among individual flies, but most bristles are organized into regular rows that are parallel and/or perpendicular to the body axis or limbs (Fig 11) [76]. Furthermore, bristles are more or less evenly spaced and aligned within each of these rows. This phenomenon has been studied since 1915 [77], and a number of models have been developed to explain the underlying mechanism of bristle formation [78–86].

Among the first models to suggest cell-cell interactions as the cause of bristle formation in *D. melanogaster* was the *“Pre-pattern Model”*. This model postulated that differential distribution of properties (such as stress points) or signals within a field of embryonic cells comprise a “prepattern” that is responsible for the organization of sensory bristles. It also suggested that each bristle comes from a group of equivalent cells, anyone of which can become a bristle. Once chosen, this cell inhibits its surrounding cells [78, 79]. Although this model summarized the macro properties of bristle formation, it did not provide any mechanistic insight into the process.

**Fig 12:**
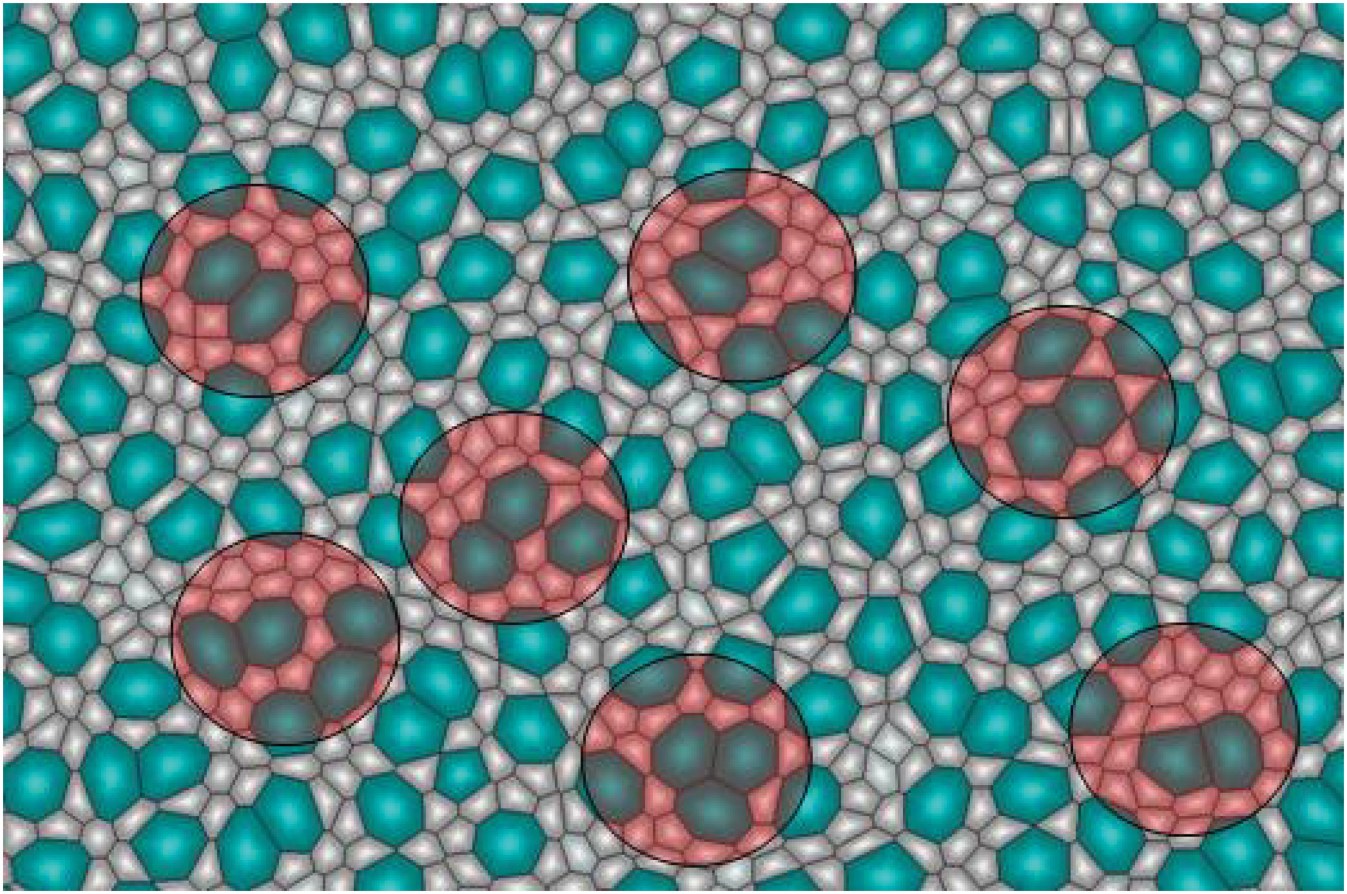
Aggregation of Bristles. The *“Pre-destined Model”* suggested that bristle sites can form based on patterns of expression of genes (*ac* and *sc* genes) [80]. Here sharp expression boundaries indicate bristle sites. Our simulation results show that the *“Pre-destined Model”* can lead to aggregation of bristles, which are not observed experimentally. For example, red circles highlight instances where ≥ 2 contacting bristle cells form aggregates.

The *“Pre-destined Model”* was the first to give mechanistic explanation for the bristle formation process. It suggests that bristle sites could be fixed based on patterns of expression of genes (*ac* and *scute (sc)* genes) [80]. Sharp expression boundaries indicate bristle sites. However, this model did not explain the lack of bristle aggregation (≥ 2 bristle cells in contact) (Fig 12).

**Fig 13:**
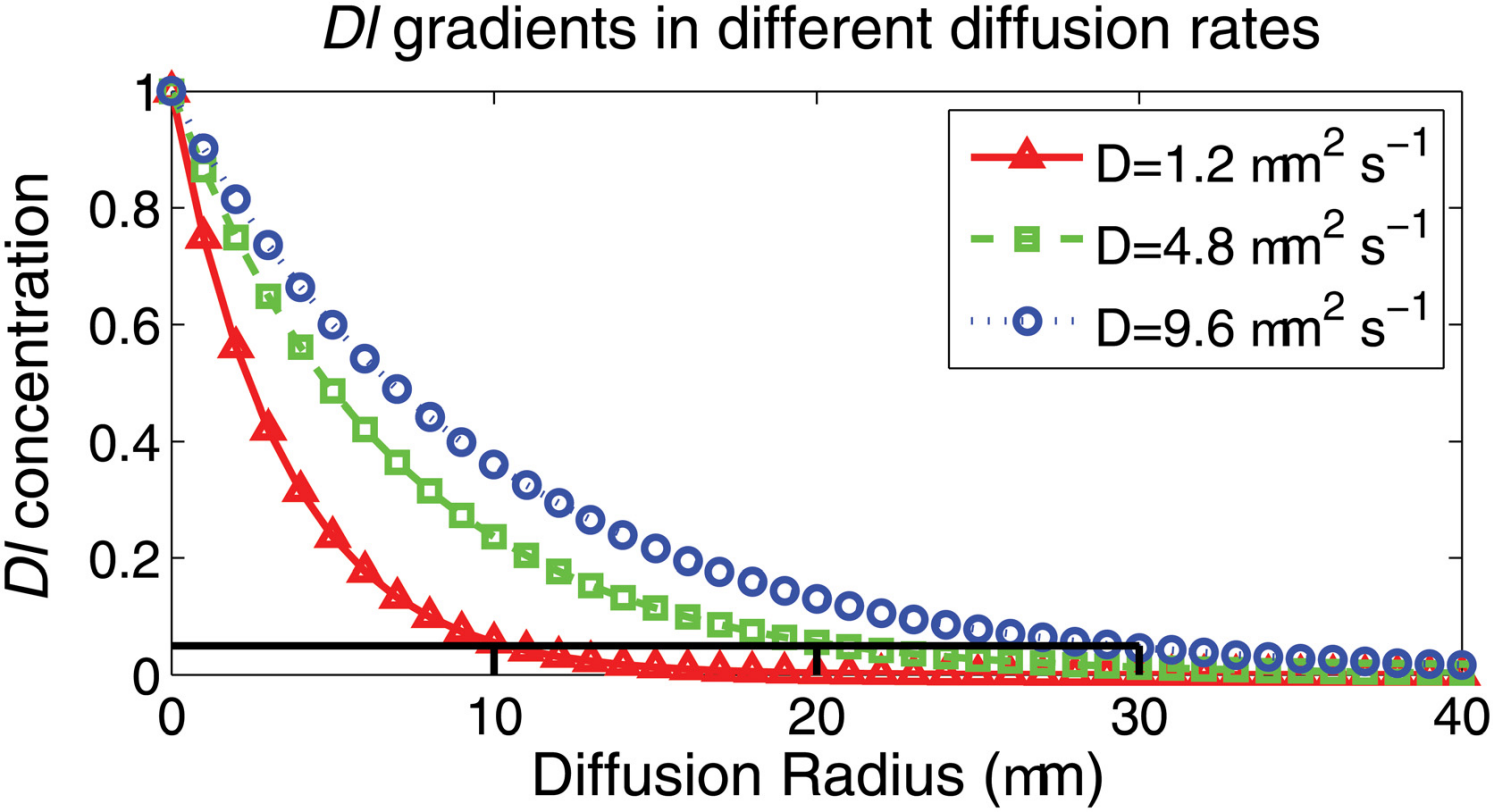
Bristle Plotting Puzzle. Simulation results of bristle pattern formation using different models. a) The pattern of gene expression used for the stripe models. Green stripes have almost equal expression of *Dl* and *N* genes but *ac* is not expressed. Blue stripes have high expression of *ac* and *Dl* genes but low expression of *N* gene. Red stripes have high expression of *ac* and *N* genes but low expression of *Dl* gene. Bristles only form in the blue stripes. b) Lateral inhibition with stripes does not ensure equal spacing or good alignment. c) Inhibition field with out stripes ensures proper spacing but does not produce a good alignment. d) Inhibition field with stripes produces equal spacing as well as good alignment.

The *“lateral inhibition”* or *“mutual inhibition”* models offered a possible mechanism to prevent aggregation of bristles [81–83]. Both postulated that the notch signaling pathway was involved in inhibiting neighboring cells to acquire similar cell fate. The discovery of the expression pattern of *ac, Dl* and *N* genes (Fig 13a) led to the modified lateral inhibition model [84, 85]. According to this model, the formation of bristles was only possible in regions that have high concentration of *ac, Dl* and low concentration of *N*. These regions form stripes, and their width was observed to be between 3 to 5 cells.

An important discovery in the field is the identification of a soluble component (Dl^*EC*^) of the Delta protein [86]. This lead to the *“inhibition field”* model, which postulates that cells can inhibit their neighbors up to a certain distance through diffusion of this soluble protein [86]. According to this model, direct cell-cell contact is not necessary. This model was also supported by the discovery of Delta-promoted filopodia that mediate long-range lateral inhibition [87], although the mechanism was quite different. Although it remains an open question whether Dl^*EC*^, filopodia, or alternatively, another soluble protein is responsible for inhibiting the cells that are not in direct contact, there is a general agreement that such a mechanism exists.

#### Inhibition Ranges based on Diffusion Coefficients

**Fig 14:**
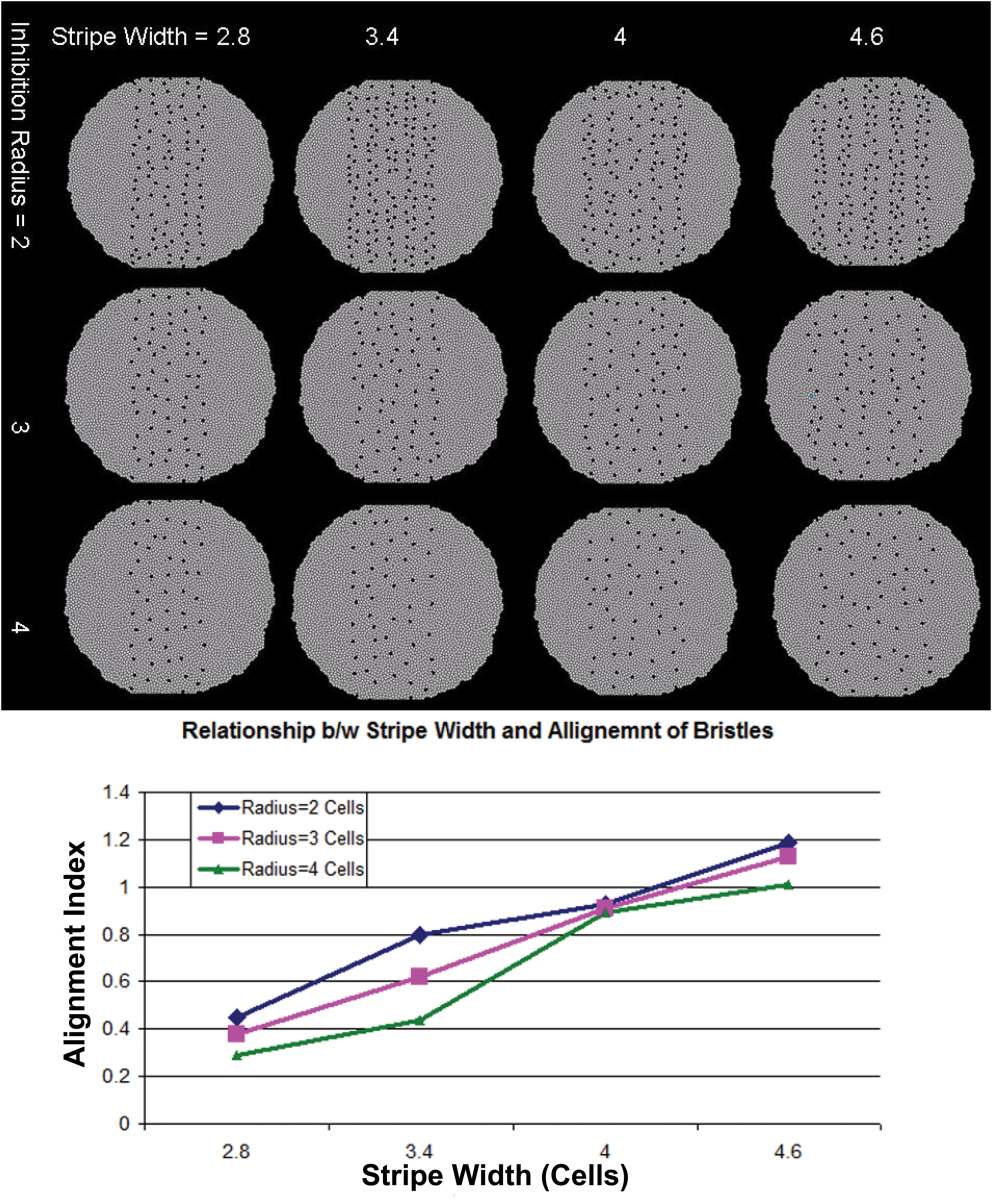
Steady state spatial gradients of *Dl* of different diffusion rates. The steady state spatial gradients of *Dl* are due to diffusion and degradation. The red solid line with triangular markers is the steady state gradient formed with the diffusion coefficient 1.2 *μm* ^2^ *s* ^−1^. The green dash line with square markers is the gradient of the diffusion coefficient 4.8 *μm* ^2^ *s* ^−1^. The blue dotted line with circle markers is of the diffusion coefficient 9.6 *μm* ^2^ *s* ^−1^. The black straight lines represents the 0.05 *Dl* concentration threshold for cellular response.

We first assume the average diameter for epithelial cells in wing imaginal disc to be *ca.* 10 *μm*[88]. The inhibition field differs when using different diffusion coefficients of *Dl* ligand. Following Merks *et al.*, we assume the *Dl* gradient is driven by both diffusion and degradation of *Dl* over the ECM [89]. Following [90], we calculate the steady state gradient of *Dl* at three different diffusion coefficients of 1.2 *μm* ^2^ *s* ^−1^, 4.8 *μm* ^2^ *s* ^−1^, and 9.6 *μm* ^2^ *s* ^−1^[91] (Fig 14). We further assume a minimum threshold of *Dl* density of 0.05 for concentration-dependent signal responses in cells, *i.e.*, cells can be activated only when the *Dl* concentration is above the dimensionless cell response threshold of 0.05 (black indicating lines in Fig 14). With the three diffusion rates tested, the farthest cells that *Dl* signal can reach are within 10*μm*, 20*μm*, and 30*μm*, respectively. This corresponds to the 1, 2, and 3 layers of neighboring cells in our simulations.

#### Simulations of Different Mechanisms and Parameter Sensitivities

We have carried out simulations to test each of the models by growing a tissue from 2 cells to about 4000 cells. The concentration of bristle formation related genes *ac, Dl* and *N* were taken from literature [80–86], these concentrations are treated as an input for the simulations (see S1 Appendix). To better understand how the formation of bristle pattern is regulated, we also analyze the parameter sensitivities of different inhibition ranges and stripe widths.

Our simulation results show that the *“Pre-destined Model (No inhibition, Dl inhibition range = 0 cell layers)”* can lead to aggregation of bristles, *i.e.* ≥ 2 contacting bristle cells form clusters, which are not observed experimentally (Fig 12). Using the *“lateral inhibition (Dl inhibition range = 1 cell layer)”* model (S1 Appendix), where each cell can inhibit 5 to 6 surrounding cells [92], we found that the fraction of bristles (≈ 16%) was much more than experimentally observed. In addition, there was no detectable alignment in regular rows. With the *“inhibition field (Dl inhibition range = 2, 3, or 4 cell layers)”* model (S1 Appendix), we found that if an inhibition radius of 3 layers of neighboring cells is used, evenly spaced bristles form (Fig 13c).

Similarly, using the *“lateral inhibition with stripes (Dl inhibition range = 1 cell layer)”* model (S1 Appendix), where each cell can inhibit 5 to 6 surrounding cells within the stripe, the fraction of bristles (≈ 8%) was much more than experimentally observed and no alignment could be detected. Therefore, the alignment and equal spacing observed on the epidermis of *D. melanogaster* cannot be reproduced by the *“lateral inhibition”* and *“lateral inhibition with stripes”* models (Fig 13b).

**Fig 15:**
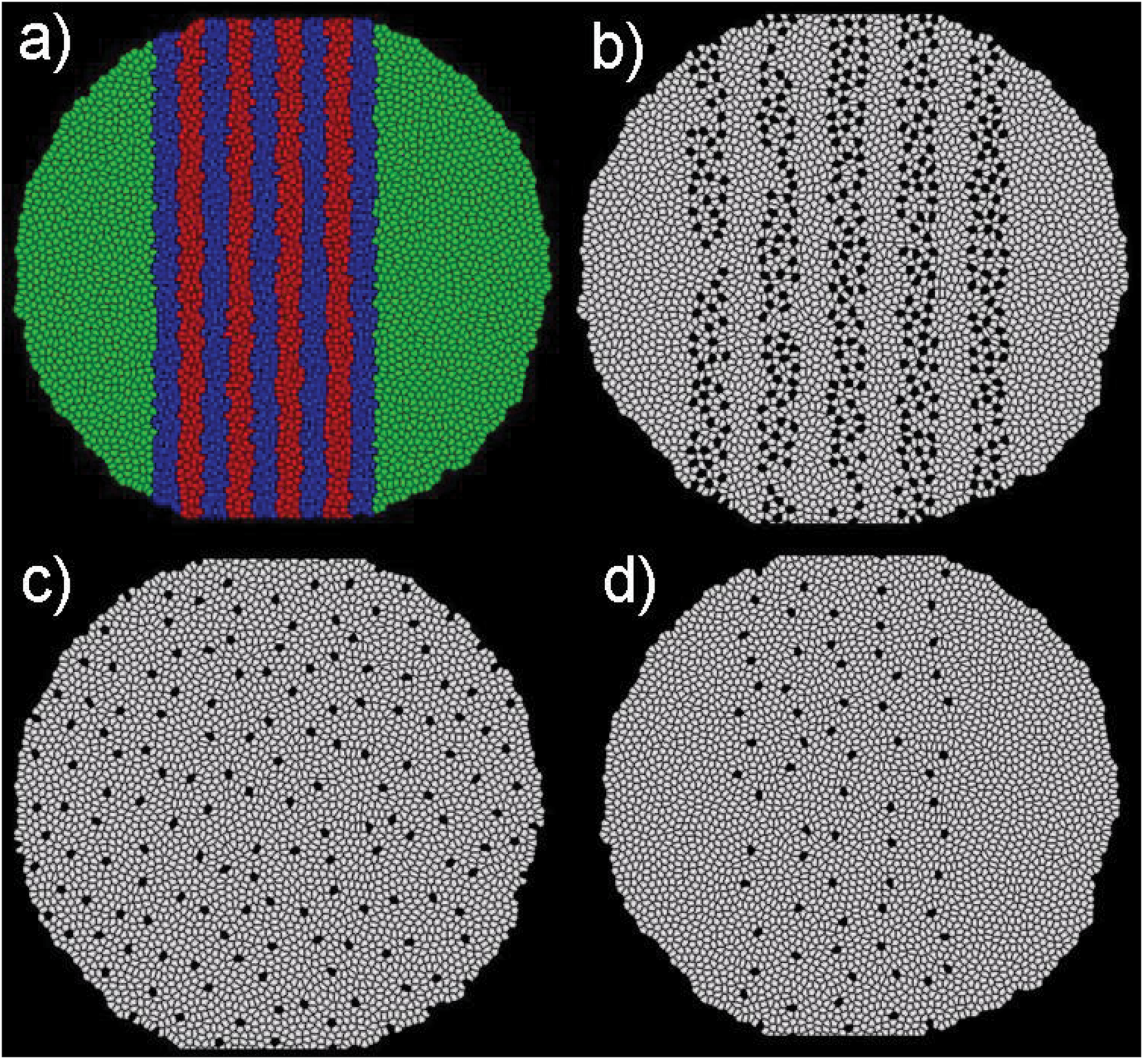
Relationship between stripe width and Inhibition Radius. Effects of stripe width and inhibition radius on the alignment of the bristles. (Top) The stripe width increase from left to right and the inhibition field radius increases from top to bottom. For a fixed Inhibition radius (horizontal rows) small stripe width produces a better alignment (Alignment Index ϱ is smaller). For a fixed stripe width (horizontal rows), large inhibition field radius produces a better alignment. (Bottom) The degree of alignment of bristle cells increases as the stripe width is reduced and inhibition radius is increased, suggesting that the alignment is directly proportional to the inhibition radius and inversely proportional to the stripe width.

We found that regular rows of bristle cells with even spacing can be produced with different choices of the stripe width and the inhibition radius (*Dl* inhibition range = 2, 3, or 4 cell layers). Through simulations, we found that the degree of alignment of bristle cells increases as the stripe width is reduced and inhibition radius is increased (Fig 15). To quantitatively analyze this relationship, we ran simulations with different combinations of stripe width and inhibition radius. Our results show that alignment is directly proportional to the inhibition radius, and inversely proportional to the stripe width (Fig 15). This is consistent with experimental observations, where increase in stripe width has been observed to cause increase in the number of bristles and their misalignment [93].

**Fig 16:**
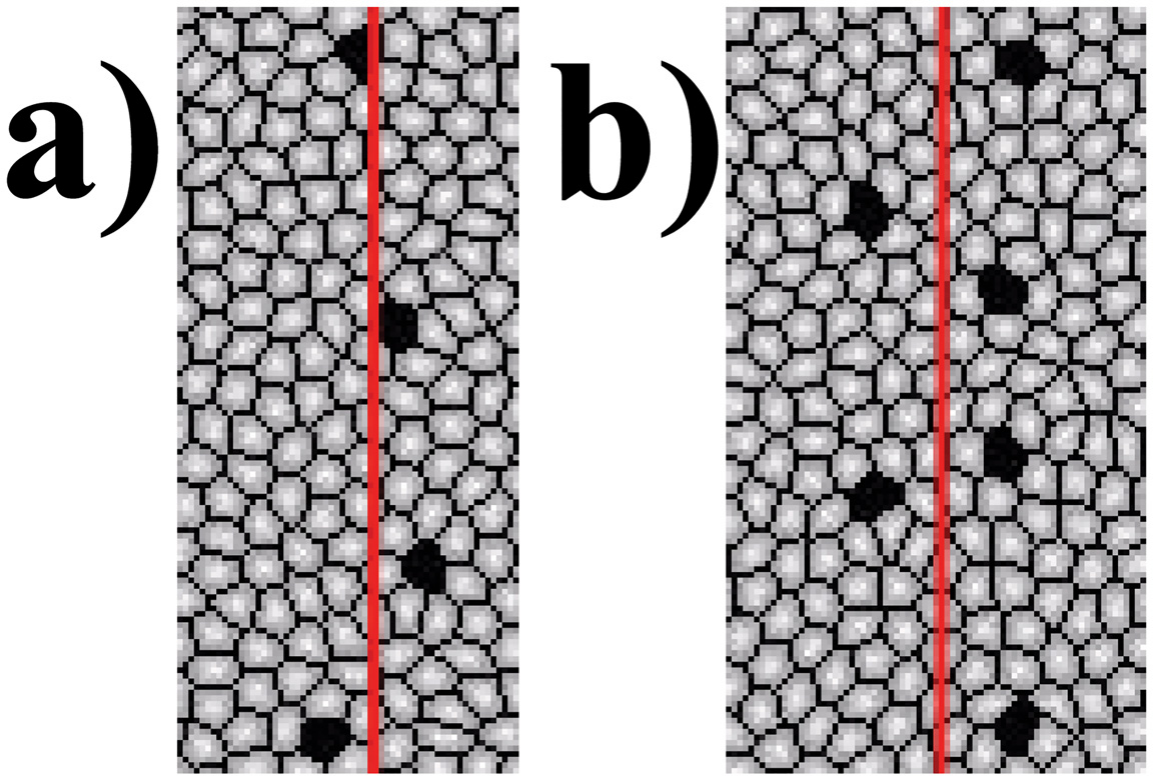
The degree of alignment by an index ϱ. It is calculated by drawing a vertical line in the middle of each stripe and summing the number of cells that lie between the bristles and the line. This is then normalized with the total number of bristles in the stripe. Here, a) is an example of good alignment with ϱ = 0.5 and b) is an example of bad alignment with ϱ = 2.0

Combining the *“inhibition field”* model with the expression pattern of *ac, Dl* and *N* in stripes (S1 Appendix), we are able to obtain evenly spaced as well as aligned regular rows of bristles, which reproduced results from experimental studies (Fig 13d) [85]. Here we quantify the degree of alignment by an index ϱ, which is calculated by drawing a vertical line in the middle of each stripe and summing the number of cells that lie on a horizontal line segment between the bristles and the vertical line. This is then normalized with the total number of bristles in the stripe (Fig 16).

$$\begin{array}{c} \varrho =\sum_{j}\sum_{i}d\left(i,j\right)/N_{j} \end{array}$$ (4)

Here *d*(*i*,*j*) is the number of cells that lie between the vertical line and bristle *i* in stripe *j*. *N*_*j*_ is the total number of bristles in stripe *j*. A value close to zero indicates perfect alignment and a large value indicates misalignment. In this case, we obtain ϱ = 0.62, with 55% of the bristles lying on the vertical line. Our simulation results using different models are summarized in Table 3.

**Table 3:** Simulation results for *“Pre-destined”*, *“Lateral inhibition”*, *“Lateral inhibition with stripes”*, *“Inhibition Field”*, and *“Inhibition Field with Stripes”* models.

Model	Characteristics	Simulation Results
Pre-destined	Based on expression of genes (*ac* and *sc*).	Leads to aggregation of bristles.
Lateral Inhibition	Inhibit neighboring cells through notch signaling pathway.	Fraction of bristles (≈ 16%) is greater than experimental observations.
Lateral Inhibition with Stripes	Bristles can be formed only in regions with high concentration of *ac, Dl* and low concentration of *N*. Bristles inhibit neighboring cells through notch signaling.	Cannot produce aligned or evenly spaced bristles. The fraction of bristles (≈ 8%) is greater than experimental observations.
Inhibition Field	Inhibit neighboring cells through notch signaling with a soluble protein or Delta-promoted filopodia.	Bristles are evenly spaced but not aligned.
Inhibition Field with Stripes	Bristles can be formed only in regions with high concentration of *ac, Dl* and low concentration of *N*. Bristles inhibit neighboring cells through notch signaling with a soluble protein or Delta-promoted filopodia.	Bristles are evenly spaced and 55% are aligned well.

### Epithelial Tissue Size Control

We now discuss the application of our method to study the fundamental problem of the homeostatic size control of epithelial tissues controlled by stem cell lineages. Epithelial tissues is one of the four basic types of animal tissues among connective, muscle and nervous tissues [94]. Precise and robust homeostatic control of tissue size is essential for tissue development. Tissue size control has been the subject of numerous studies [61, 95–97, 97–99], majority of which are based on population averages and are without detailed spatial-temporal information. At the heart of the problem is understanding the mechanism of the activation and inhibition of proliferation and differentiation of different types of cells (*i.e.*, stem cells, intermediate progenitor cells, and fully differentiated cells), which are under the influence of various feedback loops controlled by secreted factors [61, 95–98, 100]. In this example, we apply our model to study the homeostatic size control of the mammalian olfactory epithelium (OE), which is under control of stem cell lineages. We explore the effects of inhibition range of secreted factor to stem cell growth, as well as the effects of symmetric *vs* asymmetric cell divisions of stem cells. Additional detailed study of other aspects of tissue size control can be found in [101].

#### Cell Lineage and Division Types

Our cell lineage model is based on previous studies [61, 102, 103]. There are three different cell types in our model: stem cells, intermediate progenitor cells, and fully differentiated cells. Stems cells can develop into progenitor cells, and progenitor cells can develop into differentiated cells. Stem cells have unlimited ability to divide and progenitor cells can divide at most twice. Differentiated cells do not divide.

**Fig 17:**
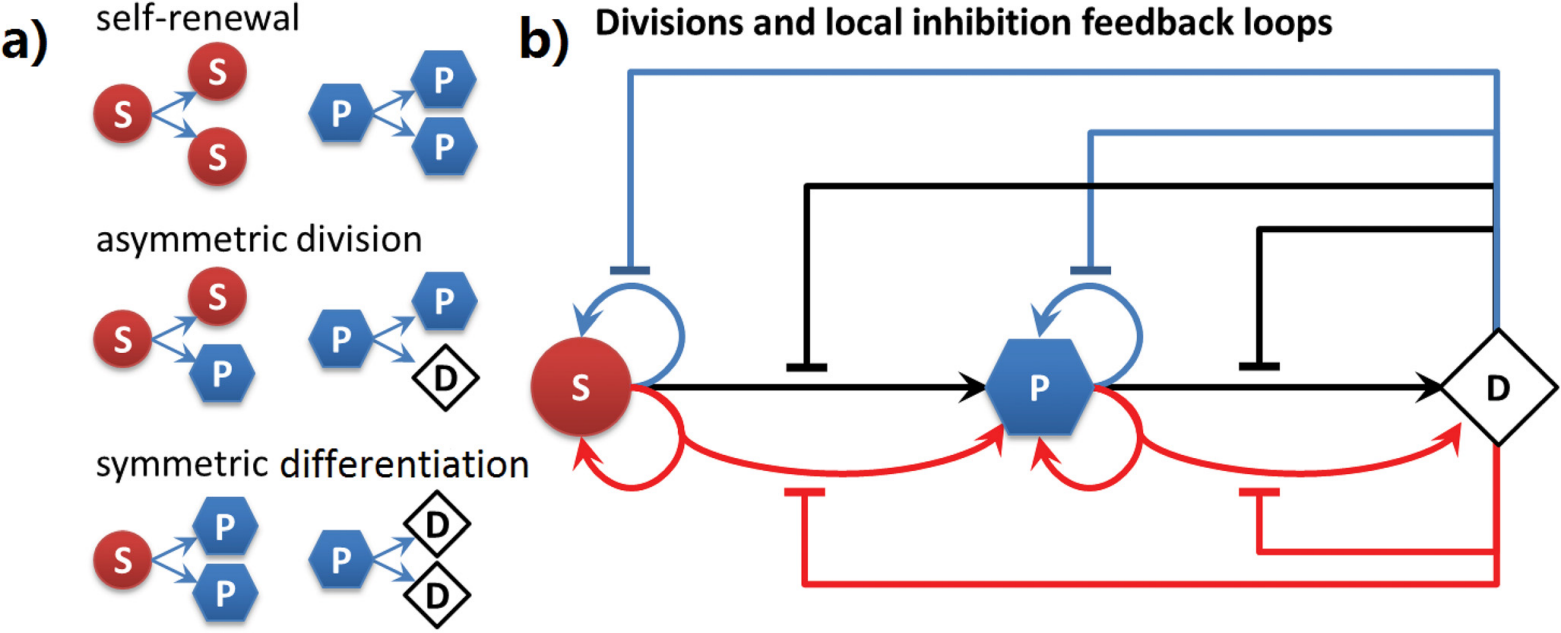
Model of feedback circuits for tissue size control. a) Division types of stem cells and progenitor cells. Red sphere labeled with (S) indicates stem cells, blue hexagon (P) indicates progenitor cells, and white diamond (D) indicates differentiated cell. The same color code is used for illustration of resulting tissues. b) Feedback controls of stem cell model. Blue arrows indicate self-renewal or proliferation divisions. Black arrows indicate symmetric differentiation divisions. Red arrows indicate asymmetric divisions. Flat-head arrows extending from differentiated cell with corresponding colors indicate inhibitions to respective type of divisions.

There are three different types of cell divisions for stem cells and progenitor cells (Fig 17A) [61, 102, 103]. In *self-renewal*, a stem cell or a progenitor cell is divided into two daughter cells of the same type as the mother cell. In *symmetric differentiation*, a stem cell and a progenitor cell are divided into two progenitor and two differentiated daughter cells, respectively. In *asymmetric division*, a stem cell is divided into two daughter cells of different types, one stem cell and one progenitor cell. Similarly, a progenitor cell is divided into two daughter cells of different type: a progenitor daughter cell and a differentiated cell. Note that as progenitor cell can maximally divide twice, the second division is always symmetric division.

#### Cellular Feedback Circuits

In our model, the growth rates of cells and the choices of division types for stem cells and progenitor cells are independently controlled by negative feedback loops involving various protein factors (Fig 17B). These are proteins secreted from differentiated cells located within a specific diffusion radius from the affected stem cell or progenitor cell.

We assume a basal cell growth rates $v_{S}^{0}$ for stem cell and $v_{P}^{0}$ for progenitor cell. In addition, the three division types are assumed to have equal basal probabilities of being chosen. For stem cell, the probability *p*_*Sr*_ for self-renewal, *p*_*Ss*_ for symmetric division, and *p*_*Sa*_ for asymmetric division all take the value of 1/3 when there are no inhibitions: $p_{Sr}^{0}=p_{Ss}^{0}=p_{Sa}^{0}=1/3.$ Similarly, the corresponding probabilities for progenitor cells are also set to 1/3 when there are no inhibitions: $p_{Pr}^{0}=p_{Ps}^{0}=p_{Pa}^{0}=1/3.$

Following [61], we use a total of eight Hill functions to model the negative feedback to the growth rates and probabilities of choosing different division type for stem cells and for progenitor cells (Eq (5)). The growth rates and probabilities at time *t* are calculated as:

$$\begin{array}{ccc} v_{S}\left(t\right) & = & \frac{v_{S}^{0}}{1+g_{S}N_{D}\left(t\right)}\;\text{for}\;\text{growth}\;\text{rate}\;\text{for}\;\text{stem}\;\text{cell,}\\ p_{Sr}\left(t\right) & = & \frac{p_{Sr}^{0}}{1+h_{Sr}N_{D}\left(t\right)}\;\text{for}\;\text{probability}\;\text{of}\;\text{stem}\;\text{cell}\;\text{self-renewal,}\\ p_{Ss}\left(t\right) & = & \frac{p_{Ss}^{0}}{1+h_{Ss}N_{D}\left(t\right)}\;\text{for}\;\text{probability}\;\text{of}\;\text{stem}\;\text{cell}\;\text{symmetric}\;\text{differentiation,}\\ p_{Sa}\left(t\right) & = & \frac{p_{Sa}^{0}}{1+h_{Sa}N_{D}\left(t\right)}\;\text{for}\;\text{probability}\;\text{of}\;\text{stem}\;\text{cell}\;\text{asymmetric}\;\text{division.}\\ v_{P}\left(t\right) & = & \frac{v_{P}^{0}}{1+g_{P}N_{D}\left(t\right)}\;\text{for}\;\text{growth}\;\text{rate}\;\text{of}\;\text{progenitor}\;\text{cell,}\\ p_{Pr}\left(t\right) & = & \frac{p_{Pr}^{0}}{1+h_{Pr}N_{D}\left(t\right)}\;\text{for}\;\text{probability}\;\text{of}\;\text{progenitor}\;\text{cell}\;\text{self-renewal,}\\ p_{Ps}\left(t\right) & = & \frac{p_{Ps}^{0}}{1+h_{Ps}N_{D}\left(t\right)}\;\text{for}\;\text{probability}\;\text{of}\;\text{progenitor}\;\text{cell}\;\text{symmetric}\;\text{differentiation,}\\ p_{Pa}\left(t\right) & = & \frac{p_{Pa}^{0}}{1+h_{Pa}N_{D}\left(t\right)}\;\text{for}\;\text{probability}\;\text{of}\;\text{progenitor}\;\text{cell}\;\text{asymmetric}\;\text{division.} \end{array}$$ (5)

Here *N*_*D*_(*t*) is the number of differentiated cells in the neighborhood within a specific number of layers of cells, which can be easily calculated using the half-edge data structure from the cell growth model. Hill parameters *g*_*S*_, *h*_*Sr*_, *h*_*Ss*_ and *h*_*Sa*_ are for growth rate, probabilities of self-renewal, symmetric and asymmetric divisions of stem cells, respectively. *g*_*P*_, *h*_*Pr*_, *h*_*Ps*_ and *h*_*Pa*_ are corresponding parameters for progenitor cells. When new probabilities of division types are generated, they are normalized so the probabilities of three division types sum to one for each cell.

Cell division happens when the cell volume doubles. The division type of a stem cell or a progenitor cell is assigned based on the calculated probabilities of division types. The growth rates and division types obtained from feedback circuits for each individual cell are then used as input to model the growth of that cell, allowing more realistic cell behavior to be simulated. The whole system of cells is therefore coupled spatio-temporally to enable modeling of tissue development with stem cell lineage.

#### Effects of inhibition range

It is well known that differentiated cells inhibit growth and division of stem cells through secreted factors [61, 98, 100]. Using a model of a mixture of different cell types, we explore how the range of inhibition affects the ability of a growing tissue to achieve size control.

**Fig 18:**
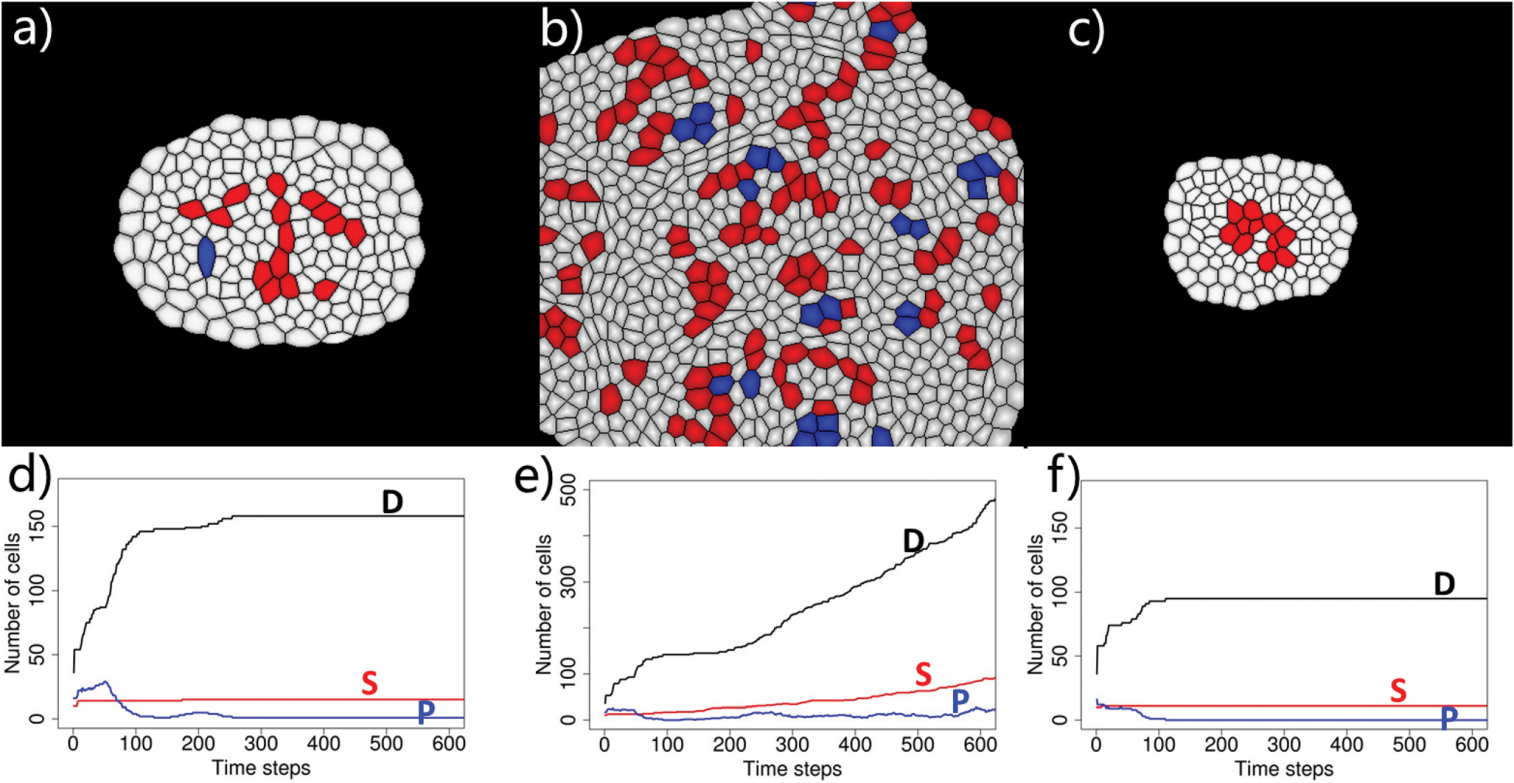
Effects of different inhibition ranges of secreted factors in negative feedback loop to stem cells on tissue size control. Examples of tissue pattern and the time course of population size of different cell types when stem cells are inhibited by differentiated cells located within (a,d) 3-, (b,e) 2-, and (c,f) 4-layers of neighboring cells, respectively. Normal size control with the ability to regenerate is achieved when 3 layers of neighboring cells are inhibited (a, and d). When 2 layers of cells are inhibited, size control is no longer possible (b and e). When 4 layers of cells are inhibited, tissue size is suppressed.

Secreted protein factors from differentiated cells may have different diffusion radius, for example, due to difference in solubility. We tested models where secreted proteins from a differentiated cell inhibit 2, 3, or 4 layers of cells in the immediate neighborhood. We carried out four independent simulations for each condition, starting from a small planar tissue of 64 cells with 10 stem cells (red) surrounded by 16 progenitor cells (blue) and 38 differentiated cells (white). Fig 18a, 18b, and 18c depict simulation results. The corresponding time curve of size of populations of stem cells (S), progenitor cells (P), and differentiated cells (D) are shown in Fig 18d, 18e, and 18f, respectively.

We assume normal tissue growth occurs when stem cells are inhibited by neighboring differentiated cells within three layers. Under this condition, tissue growth gradually slows down and eventually stops after reaching a threshold, when the homeostatic steady state is established (S3 Movie). Fig 18a shows the spatial pattern of cells in steady state. Fig 18d shows the time course of the size of different cell population. In this homeostatic steady state, differentiated cells capable of carrying out physiological functions dominate the tissue. At the same time, a small number of stem cells remain, ensuring that the potential for tissue regeneration is retained. This is also seen in experimental studies [104].

When the inhibition range is reduced to only two layers, tissue size increases monotonically with succeeding generations (Fig 18b and 18e) (S4 Movie). Size control is no longer possible. On the other hand, when the inhibition range is increased to four layers, the overall size of the tissue is suppressed, although size control is achieved (Fig 18c and 18f) (S5 Movie). Overall, our results highlight the important role of inhibition range of secreted factors. An appropriate inhibition range of secreted factors from differentiated cells in the negative control feedback loop is important for achieving tissue size control while maintaining ability of tissue regeneration.

#### Effects of inhibiting stem cell symmetric and asymmetric division

The balance between symmetric and asymmetric divisions of stem cells is critical for normal tissue development, wound healing, and tissue regeneration [105–107]. Its disruption can result in abnormal tissue development and the induction of tumor [105]. This balance depends on both intrinsic information such as cell polarity factors, as well as external signals [105].

**Fig 19:**
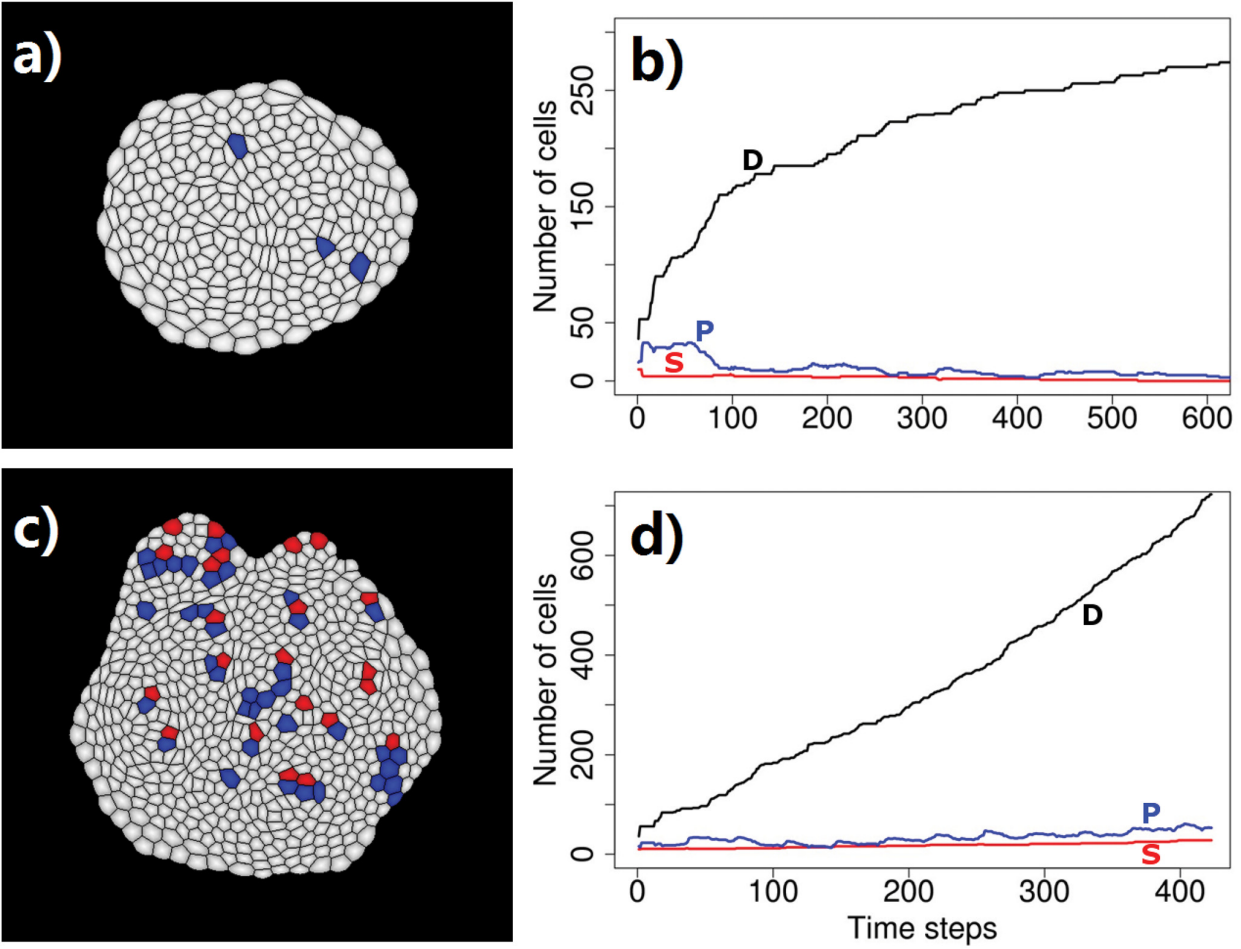
Effects of inhibitions to stem cell symmetric and asymmetric divisions. An example of tissue formation without inhibition to symmetric division is shown in a), and its corresponding time-course of the size of population of different cell types is shown in b). c) An example of tissue formation without inhibition to asymmetric division and d) the corresponding time course of size of populations of different cell types.

Our cell model can be used to study the effects of symmetric and asymmetric division in stem cells. We first explore the effects of altered symmetric differentiation of stem cells on tissue development. When the inhibition to symmetric differentiation of stem cells is removed, the pool of stem cells is quickly depleted. The tissue size at steady state is much smaller compared to that of normal development. In addition, the tissue does not maintain a population of stem cells (Fig 19a and 19b) (S6 Movie). As a result, tissue developed without inhibition to stem cell symmetric differentiation would be severely compromised in its ability for wound healing and regeneration.

Next we explore the effects of increased asymmetric division of stem cells. Asymmetric division was thought to be one of the defining characteristics of stem cells, because it can maintain a population of stem cells, at the same time producing functional differentiated cells [105]. Our results show that when the inhibition to asymmetric division is removed, the proliferation of stem cell population persists. In addition, progenitor cells are continuously produced from stem cells, regardless of the number of differentiated cells in the surrounding environment. Under these conditions, the homeostatic tissue size control is not possible (Fig 19c and 19d) (S7 Movie). In experimental studies of hyper-proliferation and malignant growth of *Drosophila melanogaster* brain tissue [108], unregulated stem cell asymmetric division was found to lead to excessive growth of the tissue and the generation of tumor. Our simulation results is in full agreement with these experimental findings.

Taken together, our simulation results suggest that a regulation mechanism that maintains a correct balance between symmetric and asymmetric divisions is important. This balance is essential for the development and maintenance of tissues. Our results are in complete agreement with experimental studies [109], in which a number of factors that regulate symmetric and asymmetric divisions were discovered [109].

## Discussion

The coordinated efforts of a large number of cells to form an organ is a complex process that is not yet fully understood. Tissue formation occurs with precision and persistence, extending beyond individuals and even generations. We do not yet have the full picture of how changes in properties of individual cells such as cell size, shape, geometry, lineage, division, growth rate, and death affect tissue formation and the whole organism. Neither do we have sufficient information on how and when cell-cell interactions become important.

To study cell pattern formation in 2D, we have presented a physical model of cell and a simulation algorithm that incorporates cell size, shape, lineage, growth rate, death rate, and different cell-cell interactions. In this work, monolayered tissue formation can be modeled using our method by following the growth process of either a single cell, or a group of cells with arbitrarily pre-arranged spatial relationship, unlike previous studies that must start with a specific pre-existing cellular pattern [24, 28, 31, 32]. The natural growth process can be modeled without the constraints from unrealistic boundary conditions and therefore do not suffer from the associated artifacts. Our method can incorporate biological properties such as different growth rates due to the effect of different growth factors. Effects of different division orientation can also be studied [110]. Furthermore, programmed cell death during the cell cycle can be incorporated. Our model represents the geometry of cells more accurately, with inner cells treated as polygons and outer cells as disc segments, as seen in *in vivo* studies [41].

In our study, we assume force equlibrium and take small step sizes in cell volume changes. We allow vertex movement relaxes and vertices reach their stationary positions. These model assumptions can be improved upon by introducing time-sepcific, cell-type specific, and/or location specific parameter values for cell and tissue mechanical proprties. In addition, more realistic growth and shrinkage parameters for different cells can also be introduced. We believe once relevant experimental measurements become available, such improvement will lead to more realistic simulations, with perhaps additional insight gained into details of cell pattern formation.

Our implementation of the model using the *Half-Edge* data structure is very efficient and robust, and provides additional benefit of no overhead with regards to time and storage in maintaining the list of neighboring cells for each cell. We are able to simulate tissues with a large number of cells (∼ 20,000) in a very short amount of time. In addition, a visualization tool has been developed that allows the user to follow the development of the tissue visually at each time step. Our software is publicly available (S1 Software).

### Comparison with Other Models

Our model differs from previous models. Compared to the center-based model [22], more detailed cell shape is explicitly included. Instead of an idealized model of sphere interactions, cell interactions are accounted for through forces due to pressure difference, surface tension, and adhesion, allowing more complex cell interactions. Many application examples given in this work would be difficult to study using the center-based model.

Our model also differs significantly from existing vertex models. First, the process of cell growth, with details of cell shapes and their changes, are modeled explicitly. Cell shape is more realistically represented, where a cell can take up the shape of a circle (the two-dimensional equivalent of sphere), a polygon, or a polygon with an arbitrary number of circular arcs as part of the cell boundary. The specific cell shape is dictated by the geometry of neighboring cells and the pressure forces they exert. In our model, boundary cells can have curved edges, with the curvature changing gradually when cell grows. Cell areas directly relate to cell sizes, and detailed incremental quasi-static changes in cell volume is followed explicitly. Contributions from changes in pressure force due to cell volume changes are also explicitly incorporated. Cell growth can be modeled independent of cell division. As a result, details of the growth process of individual cells, including creation and elimination, as well as changes in the curved boundaries, are all accounted for explicitly.

Second, our model is more general and can be used to study cell birth, initial cell division, and subsequent cell growth in detail, *e.g.*, the initial formation of cell colonies, starting from single cells. In the explicit vertex model, a sufficient number of neighboring cells is required to close cells off to form a polygon. Typically, initial conditions require the existence of a plural number of cells (*e.g.*, 16), sometimes under periodic boundary conditions [23]. Although such models work well in studying packing of epithelial cells, the process of embryogenesis starting from a single or a handful of dividing cells are difficult to model using vertex model. In contrast, birth of a single cell and its subsequent divisions can be modeled explicitly using our approach, with details of cell shapes, including curved cell boundaries, as well as the structure of cell population explicitly followed.

Third, our method can model cell death or apoptosis more realistically. In the vertex models, cell death is modeled through cell extrusion, but cell death or cell apoptosis in other situations is not accounted for. Specifically, cell extrusion is used as a surrogate for cell death, which is modeled through the T2 transition, namely, replacement of a tiny triangle/polygon by a vertex [23]. This transition is identical to the topological change of void removal we use here. However, T2 changes can occur in the vertex model without cell death. Cell apical area shrinkage due to cell division, occurrence of multiple T1 transitions, as well as multiple steps of tissue relaxations, all can lead to T2 transition, without involving actual cell death [23]. Furthermore, the occurrence of T2 transition itself does not account for all cases of cell death. As cell apoptosis is a controlled or programmed process induced by complex cellular processes, its occurrence is independent of the T2 topological changes. For example, a cell death can occur at boundary cell, and its occurrence can also create an empty hole in the tissue, instead of being replaced by the vertex shared by its original neighboring cells. These changes would be difficult to model using existing vertex models.

In our model, death of an arbitrary cell can be modeled through the underlying biological model of apoptosis. As we can model cell growth as well as cell shrinkage explicitly, and all topological changes are exhaustively accounted for, any cell in a tissue can be assigned to be programmed to apoptosize. Its demise can be followed explicitly through implementation of schemes of negative growth rates. The resulting topological changes of hole creation or void removal, which depend on the cell mechanics of the surrounding environment, are also modeled.

The method of cell simulation reported here is similar in some aspects to several general purpose simulation methods, such as the *Surface Evolver* method. For example, both methods formulate and solve the geometric and mechanical problem in a similar fashion. However, the Surface Evolver method was designed as a generic tool for finding the minimal energy surface. It is not suited for dynamic simulations of biological tissue pattern formation involving cell growth, division, and apoptosis. Although the Surface Evolver method provides interfaces for custom defined volume constraints for individual cells, modeling constantly changing cell volumes, *i.e.* those in cell growth, is not supported [111]. Therefore, it is not suited for simulating realistic cellular and tissue pattern formations during tissue development and diseases, which often involve significant amount of cell growth and divisions. In literature, Surface Evolver has been used to study biological processes under the condition of constant cell volume and fixed cell numbers, (*e.g.* 20 cells in [112], < 100 cells in [113], and 200–380 cells in [114]). All three cases have at least 2 orders of magnitude less number of cells than that our method can simulate (on a scale *e.g.* 10^4^ cells). Furthermore, to our knowledge, the Surface Evolver has not been used to study pattern formations where extensive cell growth, cell division, cell proliferation, and their integration are required, such as in tissue development of a large number of cells.

While the integration of cell growth, cell division, and cell death with effects of secreted factors is a challenging task for general purpose simulation methods such as the Surface Evolver, our method can model dynamic changes of cell shapes and topological connectedness of tissue realistically. An efficient data structure enables fast access of information and manipulation of neighboring cells in constant time. These advantages translate to greatly improve computational efficiency, such that simulating tissue pattern formation from a single cell to tens of thousands of cells can be carried out with ease.

In summary, our model has the advantage of more realistic cell shape and can model realistically cell growth, division, and apoptosis without restrictions. Furthermore, our model does not depend on any specific initial conditions nor boundary conditions. We can model geometrical and mechanical interactions among cells more realistically, and are able to model tissue pattern formation starting from 1–2 cells, with topological events such as tissue fusion fully accounted for. A full comparison to other existing methods has been summarized in Table 1.

### Pattern Formation in Epithelial Tissues

We also give examples of how our method can be used to study cellular pattern formation. Specifically, we studied the mechanism of bristle formation on the epidermis of *D. melanogaster*. We have explored the relationship between the inhibition field radius due to the solubility of Delta-like protein and the width of the stripe, in which the genes such as *ac* and *Dl* are highly expressed. Our simulation results suggest that equal spacing between cells can be achieved through inhibition field associated with a soluble protein, such as Dl^*EC*^.

**Fig 20:**
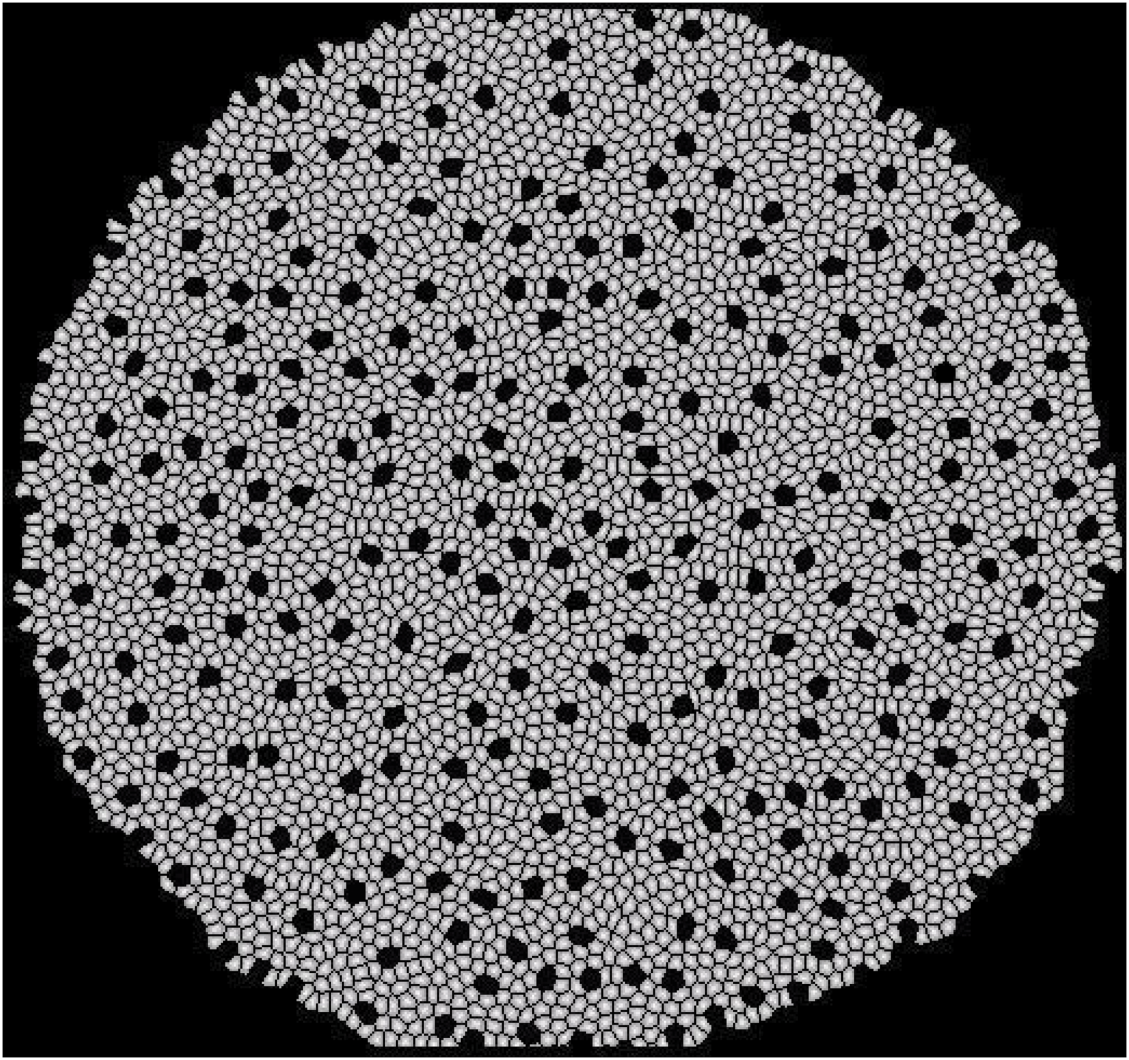
Butterfly Scales with Inhibition Field radius of 2 cells. Simulation with Inhibition field radius of 2 cells produces evenly spaced cells but regular rows do not form.

Our findings are also relevant for understanding the pattern formation of scale cells on the wings of butterfly and moth. The developmental similarity between butterfly scales and insect bristles has been known since 1896 [115]. Scale cells are arranged in parallel rows in most of the butterfly species. In most moths, they are just evenly spaced [116]. There are also several butterfly species that do not have aligned scales. Furthermore, the spacing between the scale cells varies from 1 to 5 cells in different species. It is currently unclear how these patterns have evolved. There is some evidence that the notch signaling pathway might be involved in the organization of butterfly scales [117]. Morphologically, scale cells are bigger than the surrounding cells, and are known to be able to inhibit 7–10 cells by direct cell-cell contact. Our simulation results support the idea that scale cells and bristle cells have similar underlying mechanisms for their formation. The radius of the inhibition field can vary from species to species based on the solubility of the Delta-like protein. Furthermore, our results suggest that in order to achieve good alignment, *notch/delta* like stripes are necessary (Fig 20).

### Outlook

Our model is general and has been successfully applied previously to study the effect of division plane orientation and cell rearrangements on the packing geometry of epithelial cells [110, 118], on how mechanical forces mediate localized topological changes in regulating cell topology in proliferating epithelial [110, 119], on how tissue elongation in *Drosophila* wing is regulated by oriented cell divisions, oriented mechanical forces, and reduced cell sizes [120], and on spatial population dynamics of stem cell lineage in tissue growth [101], wound healing and cancerogenesis [121].

Our model can also be extended to incorporate nutrient gradients formed by diffusion. The concentration of these nutrients can be treated as additional force acting on the cells, with stochastic effects incorporated. This model is well suited to study general spatio-temporal pattern of tissue formation such as epithelial tumor growth and the interactions between epithelial tumor cells and normal cells.

## Supporting Information

S1 Appendix Appendix. (TEX)

S1 Movie Cell apoptosis model. (WMV)

S2 Movie Tissue fusion model. (WMV)

S3 Movie Tissue size control model. (WMV)

S4 Movie Tissue size control model of 2 layers of inhibition range. (WMV)

S5 Movie Tissue size control model of 4 layers of inhibition range. (WMV)

S6 Movie Tissue size control model without inhibition to stem cell symmetric division. (WMV)

S7 Movie Tissue size control model without inhibition to stem cell asymmetric division. (WMV)

S1 Software The source code and running examples of the cell simulation software. (ZIP)
